# Comparison of Cellular Death Pathways after mTHPC-mediated Photodynamic Therapy (PDT) in Five Human Cancer Cell Lines

**DOI:** 10.3390/cancers11050702

**Published:** 2019-05-21

**Authors:** Carsten Lange, Christiane Lehmann, Martin Mahler, Patrick J. Bednarski

**Affiliations:** Department of Pharmaceutical and Medicinal Chemistry, Institute of Pharmacy, University of Greifswald, Friedrich-Ludwig-Jahn-Straße 17, 17489 Greifswald, Germany; langec@uni-greifswald.de (C.L.); lehmann.christiane92@googlemail.com (C.L.); martin-mahler@gmx.de (M.M.)

**Keywords:** mTHPC, photodynamic therapy, oxidative stress, necrosis, apoptosis, autophagy, cell cycle

## Abstract

One of the most promising photosensitizers (PS) used in photodynamic therapy (PDT) is the porphyrin derivative 5,10,15,20-tetra(*m*-hydroxyphenyl)chlorin (mTHPC, temoporfin), marketed in Europe under the trade name Foscan^®^. A set of five human cancer cell lines from head and neck and other PDT-relevant tissues was used to investigate oxidative stress and underlying cell death mechanisms of mTHPC-mediated PDT in vitro. Cells were treated with mTHPC in equitoxic concentrations and illuminated with light doses of 1.8–7.0 J/cm^2^ and harvested immediately, 6, 24, or 48 h post illumination for analyses. Our results confirm the induction of oxidative stress after mTHPC-based PDT by detecting a total loss of mitochondrial membrane potential (Δ*ψ*_m_) and increased formation of ROS. However, lipid peroxidation (LPO) and loss of cell membrane integrity play only a minor role in cell death in most cell lines. Based on our results, apoptosis is the predominant death mechanism following mTHPC-mediated PDT. Autophagy can occur in parallel to apoptosis or the former can be dominant first, yet ultimately leading to autophagy-associated apoptosis. The death of the cells is in some cases accompanied by DNA fragmentation and a G_2_/M phase arrest. In general, the overall phototoxic effects and the concentrations as well as the time to establish these effects varies between cell lines, suggesting that the cancer cells are not all dying by one defined mechanism, but rather succumb to an individual interplay of different cell death mechanisms. Besides the evaluation of the underlying cell death mechanisms, we focused on the comparison of results in a set of five identically treated cell lines in this study. Although cells were treated under equitoxic conditions and PDT acts via a rather unspecific ROS formation, very heterogeneous results were obtained with different cell lines. This study shows that general conclusions after PDT in vitro require testing on several cell lines to be reliable, which has too often been ignored in the past.

## 1. Introduction

Photodynamic therapy (PDT) is a method for the medical treatment of solid tumors based on the generation of reactive oxygen species (ROS) after photoactivation of a photosensitizer (PS). The PS is excited by light of a certain wavelength and then transfers its energy to triplet oxygen to form highly reactive singlet oxygen (^1^O_2_) or generates other ROS via a direct electron transfer to biological substrates, e.g., components of the cellular membrane. The ROS formed lead to oxidative stress within the cells of the treated tumor and eventually cause cellular death [1,2]. Thereby, a certain degree of selectivity can be achieved by restricting the illumination area to the site of the tumor. Further advantages over established cancer therapies include nontoxicity in the absence of light, the possibility of repeated treatments without cumulative toxicity, no carcinogenicity, and the fact, that PDT is a non- or only minimally-invasive approach [1,3].

One of the most promising PS used in PDT is the porphyrin derivative 5,10,15,20-tetra(*m*-hydroxyphenyl)chlorin (mTHPC, temoporfin), which was developed in 1989 [4] and approved under the trade name Foscan^®^ in 2001 in Europe for the treatment of head and neck cancers [5,6]. mTHPC displays many of the requirements for ideal PS, e.g., chemical purity, a light absorption maximum within the optical window of λ = 650–850 nm, minimal dark toxicity, and reduced photosensitivity as well as enhanced phototoxic effects compared to the first approved and still widely used PS Photofrin [7,8]. Although there is supportive data from numerous studies for the use of mTHPC in the treatment of head and neck cancers [5,9,10,11] and other cancer types [12,13], Foscan^®^ is still not approved by the FDA in the USA. Many of the in vitro data gathered over the past decades have been based on single cell line analyses, which in turn were sometimes derived from PDT-irrelevant cell sources like the biliary system, brain, bone, liver, and breast tissue, or blood, which are rather unlikely to be targeted by PDT [6,14,15,16,17].

Here, we used a panel of five human cancer cell lines from PDT-relevant tissues, i.e., lung (A-427), oral cavity (BHY), esophagus (KYSE-70), bladder (RT-4), and cervix (SISO), to investigate the underlying cell death mechanisms after mTHPC-based PDT in vitro and to compare the outcome of this treatment between cell lines under equitoxic conditions. Similarities and differences in various biological outcomes were observed between cell lines, indicating that single cell line analysis is not enough for the evaluation of in vitro effects after mTHPC-PDT. Our data suggest that there is not one specific sequence of cell death events induced by mTHPC-PDT that is common to all cancer cell types. Therefore, not only is a multiple cell line analysis required for the evaluation of PDT in vitro, but also individual protocols for specific cancer types might enhance overall phototoxic effects and success of a mTHPC-mediated PDT.

## 2. Results

### 2.1. Loss of Cellular Viability after mTHPC-PDT

**Figure 1:**
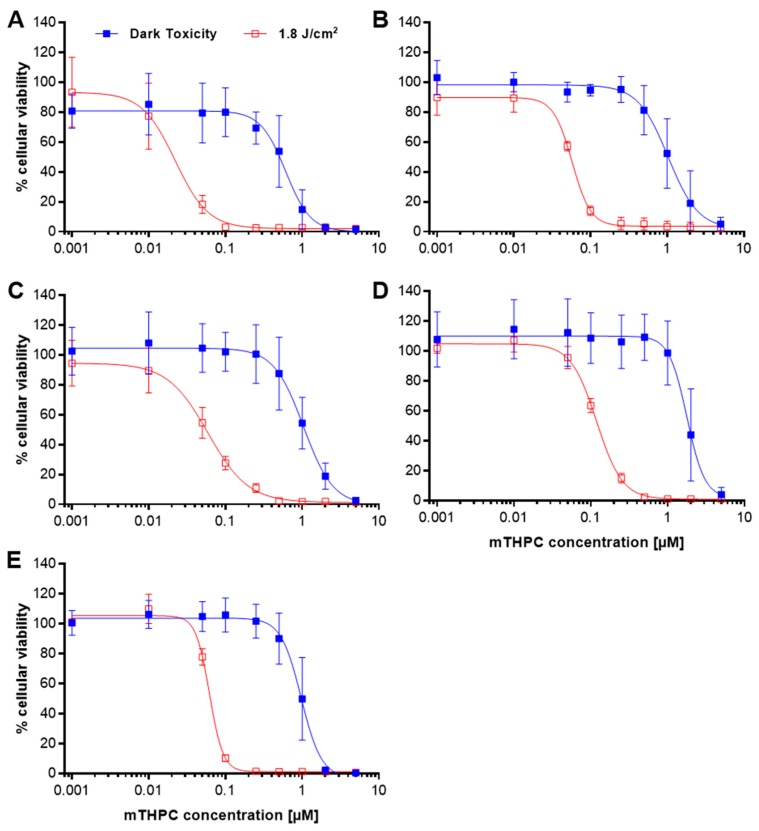
Dark (■) and light-induced (□) loss of cellular viability after mTHPC treatment as assessed by MTT viability assay. The five cell lines A-427 (**A**), BHY (**B**), KYSE-70 (**C**), RT-4 (**D**), and SISO (**E**) were treated with mTHPC for 24 h in concentrations ranging from 0.001–5.0 µM and kept in the dark (dark toxicity) or illuminated with a light dose of 1.8 J/cm^2^ (light-induced toxicity). MTT assay was carried out 24 h post illumination and the absorbance of the reduced formazan was measured at λ = 570 nm. The percentage of cell viability was calculated by dividing the absorbance for the treated group by the absorbance in the solvent dark control. IC_50_ and IC_90_ values were calculated by using Prism 6. Results are presented as means ± SD from at least three independent experiments.

The effects of mTHPC on the cellular viability of five human cancer cell lines revealed differences in the sensitivities of the cells against the PS. Figure 1A–E shows dark and light-induced cytotoxic effects of mTHPC 24 h after illumination with a light dose of 1.8 J/cm^2^ in the MTT cell viability assay. The established IC_50_ and IC_90_ values (concentrations, where 50% and 90% of the measured effect, i.e., loss of cellular viability, was observed) are summarized in Table 1. Post illumination, IC_50_ values ranged from 0.02–0.10 µM with the A-427 lung cell line being the most sensitive and the RT-4 bladder cell line being the most robust. At a concentration of 0.001 µM, cellular viability for all cell lines was ≥90% compared to a solvent-treated control (SC). With increasing concentrations, cellular viability dropped to <6% at concentrations higher than 0.5 µM (0.1 µM for A-427) for all cell lines. IC_90_ values ranged from 0.1–0.3 µM with RT-4 cells being the most robust again.

In the absence of light, IC_50_ values shifted to a higher concentration range of 0.6–1.8 µM with cellular viability ≥90% (≥70% for A-427) at concentrations as high as 0.25 µM. Cellular viability dropped to <6% only at concentrations as high as 5.0 µM (2.0 µM for A-427) for all cell lines. IC_90_ values ranged between 1.4–3.2 µM without illumination of the cells.

The established IC_50_ and IC_90_ values from the MTT data were used in further analyses of phosphatidylserine externalization, cell cycle distribution, mitochondrial membrane potential and western blot analyses of caspase 3 (cas 3), poly(ADP-ribose) polymerase (PARP), and microtubule-associated protein light chain 3 (LC3-II) for a treatment with equitoxic concentrations that allowed for comparison between cell lines.

### 2.2. Only Minor Induction of Necrosis after mTHPC-PDT

To investigate the role of necrosis as a part of the death mechanism after mTHPC-based PDT, release of lactate dehydrogenase (LDH) was measured after photodynamic treatment. LDH release is a classic assay for estimating damage to cell membranes, which is characteristic of necrosis [18]. Treatment with Triton X-100 resulted in cytotoxicity (see Table 2).

**Figure 2:**
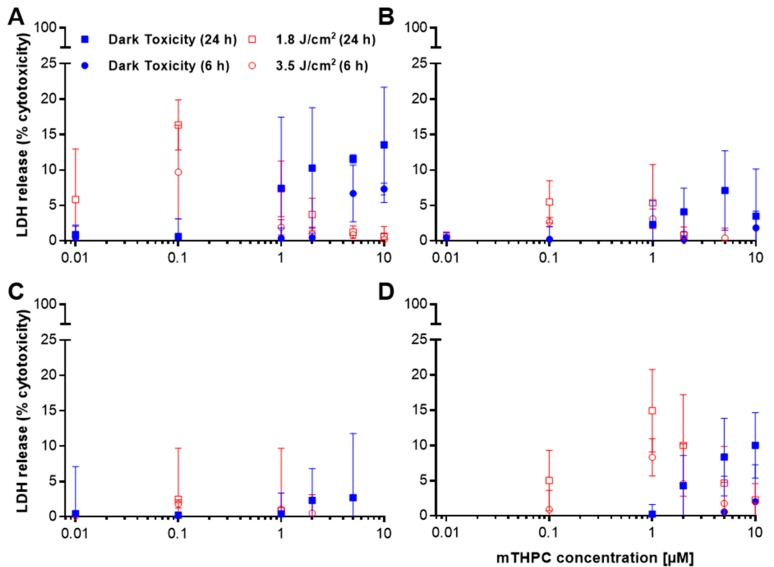

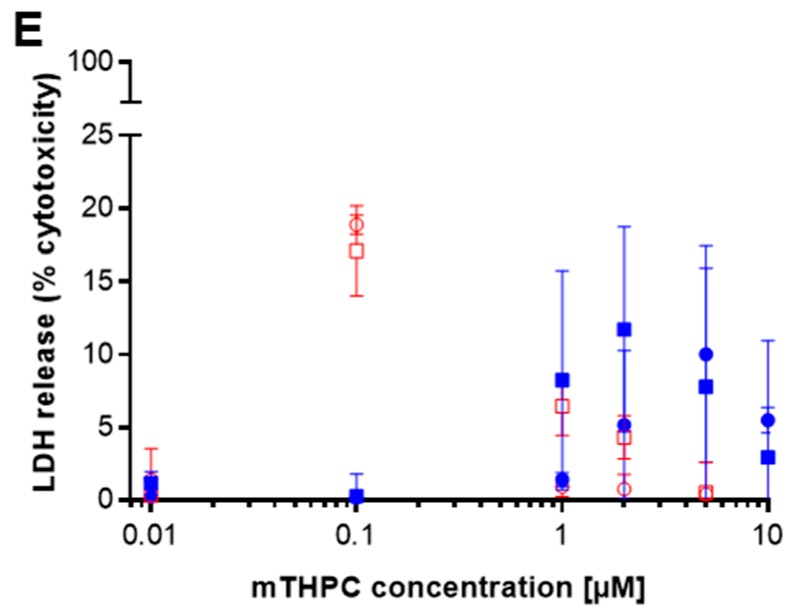
Dark (●, 6 h; ■, 24 h) and light-induced (○, 6 h; □, 24 h) cytotoxicity after mTHPC-PDT as assessed by LDH release assay. The five cell lines A-427 (**A**), BHY (**B**), KYSE-70 (**C**), RT-4 (**D**), and SISO (**E**) were treated with mTHPC for 24 h in concentrations ranging from 0.01–10.0 µM and kept in the dark (dark toxicity) or illuminated with a light dose of 1.8 J/cm^2^ or 3.5 J/cm^2^ (light-induced toxicity). LDH release assay was carried out 24 or 6 h post illumination, respectively, and the absorbance of the reduced INT was measured at λ = 490 nm. The percentage of cytotoxicity was calculated by dividing the absorbance for the treated group by the absorbance at maximum LDH release. Results are presented as means ± SD from at least three independent experiments.

The detected % cytotoxicity after treatment of five different cell lines with mTHPC did not exceed values >18.9% (at 0.1 µM mTHPC with 3.5 J/cm^2^ 6 h after PDT in SISO cells) under any experimental conditions (Figure 2A–E). In general, LDH release was higher 24 h after illumination with a light dose of 1.8 J/cm^2^ compared to cells incubated in the dark or analyzed 6 h after illumination with a higher light dose of 3.5 J/cm^2^. LDH release was highest for A-427, RT-4, and SISO cells with peak values of 16.4%, 14.9%, and 17.1% cytotoxicity 24 h post illumination. Remarkably, these values were not observed at the highest, but at low or medium concentrations of mTHPC between 0.1–1.0 µM. The highest detected % cytotoxicity for BHY and KYSE-70 were 7.1% and 2.7% within the same concentration range.

### 2.3. Oxidative Stress and Apoptotis Induction After mTHPC-PDT

#### 2.3.1. Increased ROS Generation after mTHPC-PDT

**Figure 3:**
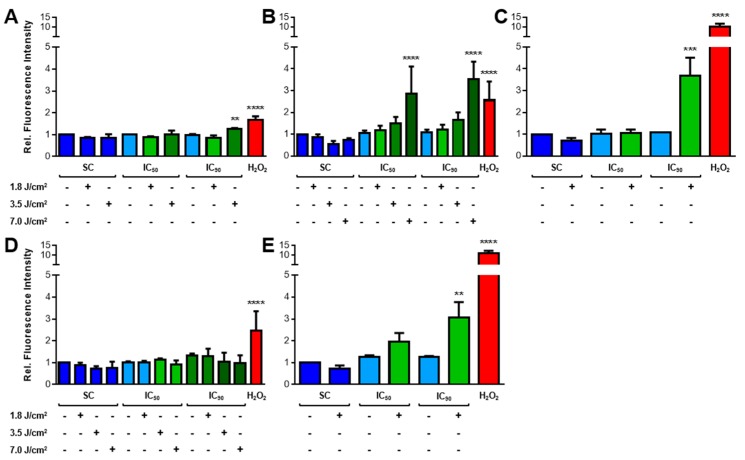
Reactive oxygen species (ROS) formation after mTHPC-PDT in A-427 (**A**), BHY (**B**), KYSE-70 (**C**), RT-4 (D), and SISO (**E**) cells. Cells were treated with solvent (SC) or equitoxic concentrations (IC_50_ or IC_90_ in Table 1) of mTHPC between 0.02–0.3 µM, illuminated with 1.8 J/cm^2^, 3.5 J/cm^2^, and 7.0 J/cm^2^ or left in the dark, stained with H_2_DCF-DA and DCF fluorescence intensity was measured directly after illumination. Flow cytometric analysis of the single cell population was carried out using the FITC channel (λ_Ex/Em_ = 488/525–550 nm). Fluorescence intensity was plotted with reference to non-illuminated, solvent-treated cells (fluorescence intensity was set to 1.0). Cells treated with 1.0–2.0 mM H_2_O_2_ for 10 min were used as positive control. Data presented as means ± SD from at least three independent experiments. (** *p* < 0.01; *** *p* < 0.001; **** *p* < 0.0001).

**Figure A1:**
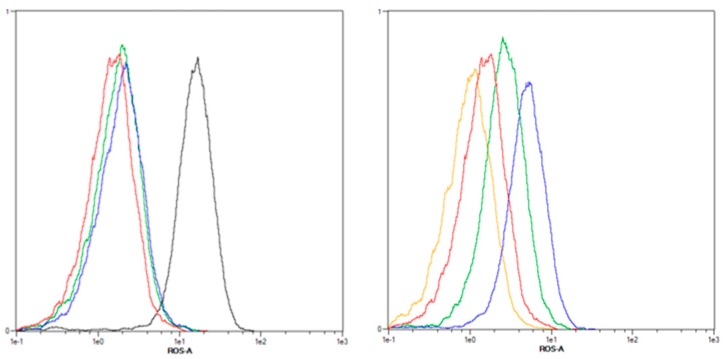
Representative flow cytometric analysis of ROS formation in SISO cells. Cells were treated with solvent (red and yellow), H_2_O_2_ (black), and the IC_50_ (green) or IC_90_ (blue) of mTHPC. Afterwards, cells were kept in the dark (left) or were illuminated with 1.8 J/cm^2^ (right) and stained with H_2_DCF-DA directly after illumination. For each sample, 10,000 events were counted and gated for the single cell population (not shown). DCF fluorescence was detected with the FITC channel (λ_Ex/Em_ = 488/525–550 nm) of a MACS Quant flow cytometer. Data were analyzed with the MACS Quantify Software.

The formation of ROS during oxidative stress is known to cause cellular death after PDT [1,2]. ROS was detected by flow cytometric analysis of DCF fluorescence intensity after staining with 2′,7′-dichlorodihydrofluorescein diacetate (H_2_DCF-DA) (see Figure A1 in Appendix A for representative analysis data). Fluorescent 2′,7′-dichlorofluorescein (DCF) is formed after contact with ROS, except singlet oxygen (^1^O_2_) [19], and the fluorescence intensity is proportional to ROS levels [20,21]. The results of intracellular ROS analyses are shown in Figure 3A–E. Direct treatment with H_2_O_2_ for 10 min led to increases of DCF fluorescence intensity. Solvent-treated cells that were kept in the dark served as the reference sample (fluorescence intensity for this sample was set to 1.0). No elevated ROS levels were observed after treatment with mTHPC in the absence of light or in solvent-treated and illuminated cells.

After mTHPC-PDT, elevated ROS levels were observed, but different mTHPC concentrations and light doses were required to increase ROS formation among the cell lines. After treatment with the IC_90_ of mTHPC and PDT with the lowest light dose of 1.8 J/cm^2^, a 3.7-fold and a 3.1-fold increase in ROS levels were observed in KYSE-70 and SISO cells, respectively, but a higher light dose of 3.5 J/cm^2^ was necessary for A-427 cells (1.3-fold increase). For BHY cells, a very high light dose of 7.0 J/cm^2^ was required to produce a significantly generation of ROS (2.9-fold, IC_50_, and 3.5-fold, IC_90_) of mTHPC. No significant increase in ROS formation was detected in RT-4 cells for any of the tested concentrations and light doses.

#### 2.3.2. Lipid Peroxidation (LPO) Only Plays a Minor Role after mTHPC-PDT

**Figure 4:**
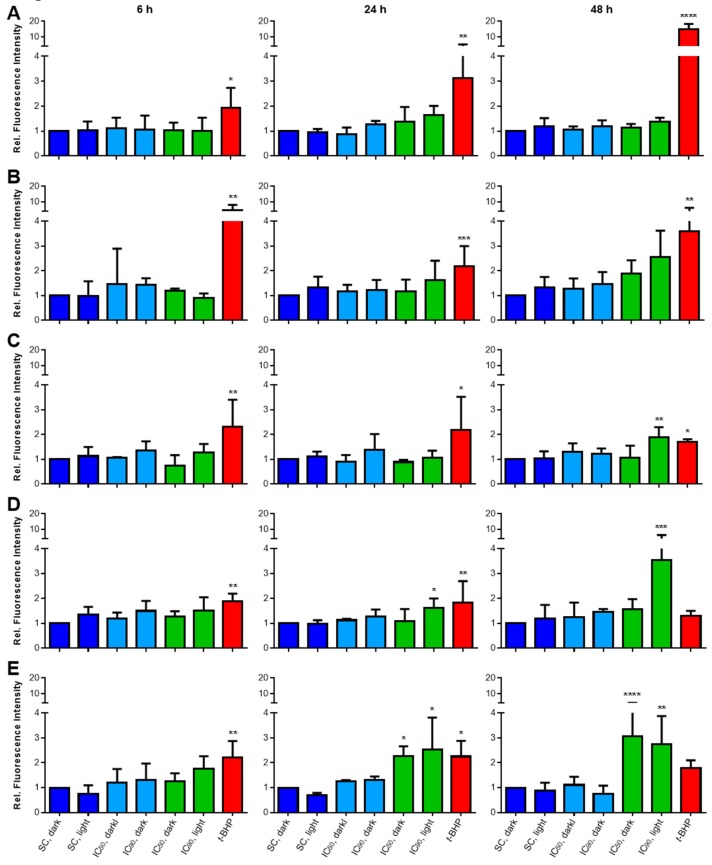
Analysis of LPO after mTHPC-PDT in A-427 (**A**), BHY (**B**), KYSE-70 (**C**), RT-4 (**D**), and SISO (**E**) cells. Cells were treated with solvent (SC) or equitoxic concentrations (IC_50_ or IC_90_ in Table 1) of mTHPC between 0.02–0.3 µM, illuminated with 1.8 J/cm^2^ or left in the dark, stained with BODIPY^665/676^ and fluorescence intensity was measured 6 (left), 24 (middle), or 48 h (right) after illumination. Flow cytometric analysis of the single cell population was carried out using the APC channel (λ_Ex/Em_ = 635/655–730 nm). Fluorescence intensity was plotted with reference to non-illuminated, solvent-treated cells (fluorescence intensity was set to 1.0). Cells treated with 0.4–3.0 mM *t*-BHP for 24 h (A-427: 0.2–0.4 mM *t*-BHP for 4 h) were used as positive control. Data presented as means ± SD from at least three independent experiments. (* *p* < 0.05; ** *p* < 0.01; *** *p* < 0.001; **** *p* < 0.0001).

**Figure A2:**
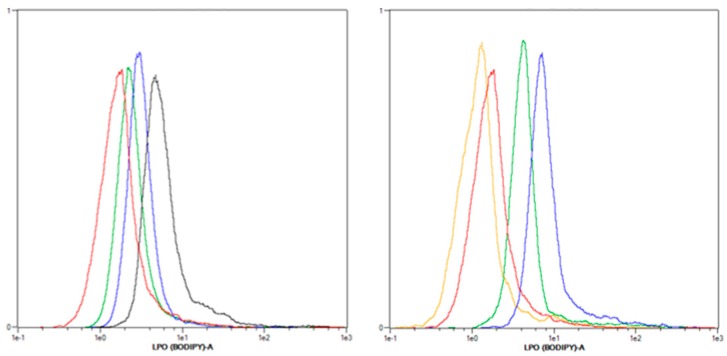
Representative flow cytometric analysis of LPO in SISO cells. Cells were treated with solvent (red and yellow), *t*-BHP (black), and the IC_50_ (green) or IC_90_ (blue) of mTHPC. Afterwards, cells were kept in the dark (left) or illuminated with 1.8 J/cm^2^ (right) and stained with BODIPY^665/676^ 24 h after illumination. For each sample, 10,000 events were counted and gated for the single cell population (not shown). BODIPY^665/676^ fluorescence was detected with the APC channel (λ_Ex/Em_ = 635/655–730 nm) of a MACS Quant flow cytometer. Data were analyzed with the MACS Quantify Software.

The detection of lipid peroxidation (LPO) was done with a flow cytometer after staining with the LPO sensor BODIPY^665/676^ (see Figure A2 in Appendix A for representative analysis data). The dye, which localizes in the cellular membrane, is oxidized upon contact with hydroxyl (OH^•^), alkoxyl (RO^•^), and peroxyl radicals (ROO^•^), leading to a change in the fluorescence spectrum [22,23]. The results of LPO analyses are shown in Figure 4A–E. Treatment with *tert*-butyl hydroperoxide (*t*-BHP) as a positive control led to more LPO in all cell lines. Solvent-treated, non-illuminated cells served as the reference sample (fluorescence intensity for this sample was set to 1.0). No enhanced formation of LPO was observed after treatment with mTHPC in the absence of light or in solvent-treated and illuminated cells in any cell line and at any time point.

Likewise, 6 h after mTHPC-PDT with a light dose of 1.8 J/cm^2^, no increased LPO occurred in any cell line. At a later time of 24 h post PDT, significantly more LPO was detected only in RT-4 (1.6-fold, IC_90_) and SISO cells (2.3–2.5-fold with both concentrations). These values were further increased 48 h after PDT in both cell lines (RT-4: 3.5-fold, IC_90_ and SISO: 2.7–3.1-fold with both concentrations). At 48 h, an increase in LPO also occurred in BHY (2.5-fold, IC_90_) and KYSE-70 cells (1.9-fold, IC_90_). No changes in LPO levels occurred in A-427 cells.

#### 2.3.3. Total Loss of Mitochondrial Membrane Potential (δψM) after mTHPC-PDT

**Figure 5:**
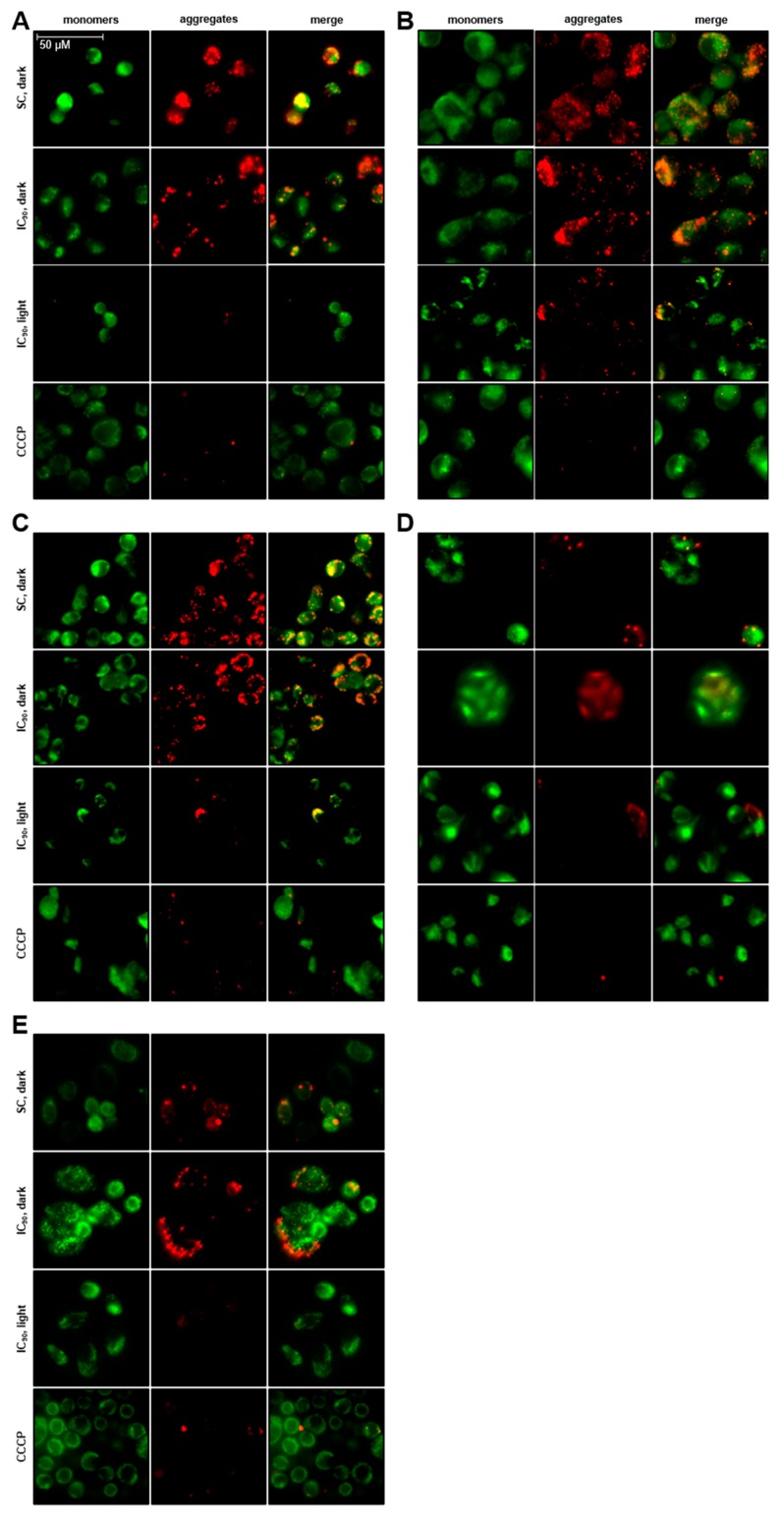
Evaluation of mitochondrial membrane potential (Δ*ψ*_m_) after mTHPC-PDT in A-427 (**A**), BHY (**B**), KYSE-70 (**C**), RT-4 (**D**), and SISO (**E**) cells. Cells were treated with solvent (SC) or equitoxic concentrations of mTHPC between 0.07–0.3 µM (equal to the respective IC_90_ in Table 1), illuminated with 1.8 J/cm^2^ or left in the dark, and stained with the cationic dye JC-1 after an incubation period of 6 h post illumination or incubation in the dark. JC-1 monomers and aggregates were visualized with a fluorescence microscope equipped with a 63× oil/1.4 NA objective. JC-1 aggregates within active mitochondria are shown in red, whereas cytosolic JC-1 monomers display a green fluorescence. A decrease in red fluorescence indicates a decline in Δ*ψ*_m_, which is a sign of early apoptosis. Fluorescence images were captured with the FITC filter cube (green; λ_Ex/Em_ = 460–500/512–542 nm) and the RHOD filter cube (red; λ_Ex/Em_ = 541–551/565–605 nm). Solvent-treated and non-illuminated cells served as the negative control with active mitochondria. As a positive control, cells were treated with 50 µM CCCP, a mitochondrial oxidative phosphorylation uncoupling agent.

To evaluate the effects of mTHPC-PDT on mitochondrial membrane potential (Δ*ψ*_m_), a widely used procedure was applied for staining the mitochondrion with the cationic dye JC-1. Green fluorescent JC-1 monomers and red fluorescent JC-1 aggregates were visualized by fluorescence microscopy. A decrease in JC-1 polymer aggregation within mitochondria indicates a decline in Δ*ψ*_m_, which is a characteristic sign of oxidative stress and apoptosis induction [24,25]. Cytosolic JC-1 monomers (green) were observed in all cell lines irrespective of the treatment 6 h after PDT or incubation in the dark (Figure 5A–E). High amounts of JC-1 polymers (red) were observed in all cell lines after treatment with solvent or the IC_90_ of mTHPC in the absence of light, indicating the presence of active mitochondria with normal Δ*ψ*_m_. As a positive control, cells treated carbonyl cyanide *m*-chloro phenyl hydrazone (CCCP) were used and showed a complete loss of red fluorescence.

After treatment with mTHPC and subsequent application of light, a decrease in red fluorescence was observed in all cell lines. These results indicate that mTHPC-PDT leads to a depolarization of the Δ*ψ*_m_ and a loss of mitochondrial activity, which is a sign of early apoptosis.

#### 2.3.4. Induction of Phosphatidylserine Externalization after mTHPC-Mediated PDT

**Figure 6:**
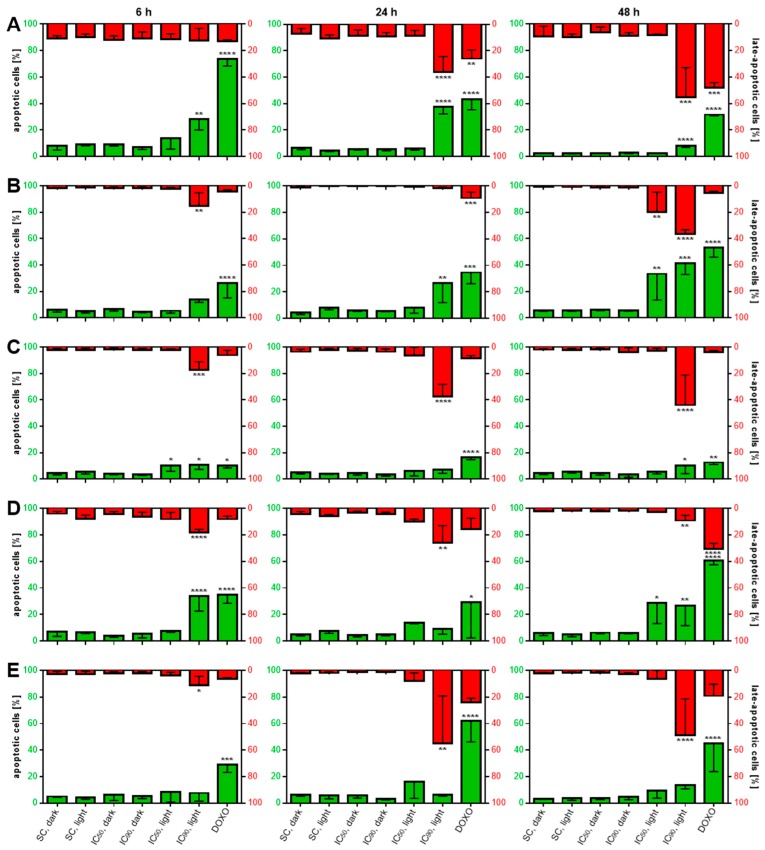
Induction of apoptosis, measured by phosphatidylserine externalization after mTHPC-PDT in A-427 (**A**), BHY (**B**), KYSE-70 (**C**), RT-4 (**D**), and SISO (**E**) cells. Cells were treated with either solvent (SC) or equitoxic concentrations (IC_50_ or IC_90_ in Table 1) of mTHPC between 0.02–0.3 µM, illuminated with 1.8 J/cm^2^ or left in the dark, stained with Annexin V-FITC and propidium iodide (PI) and analyzed 6 (left), 24 (middle), or 48 h (right) after photodynamic treatment. Flow cytometric analysis of the single cell population was carried out using the FITC channel (λ_Ex/Em_ = 488/525–550 nm) for the detection of Annexin V-positive cells and the PI channel (λ_Ex/Em_ = 488/655–730 nm) for PI-positive cells. The percentage of apoptotic cells is plotted on the left axis, while late-apoptotic cells can be seen on the right axis in opposite direction. Non-illuminated, solvent-treated cells served as the reference sample; cells treated with 0.5–5.0 µM DOXO were used as positive control. Data presented as means ± SD from at least three independent experiments. (^*^ *p* < 0.05; ^**^ *p* < 0.01; ^***^ *p* < 0.001; ^****^ *p* < 0.0001).

**Figure A3:**
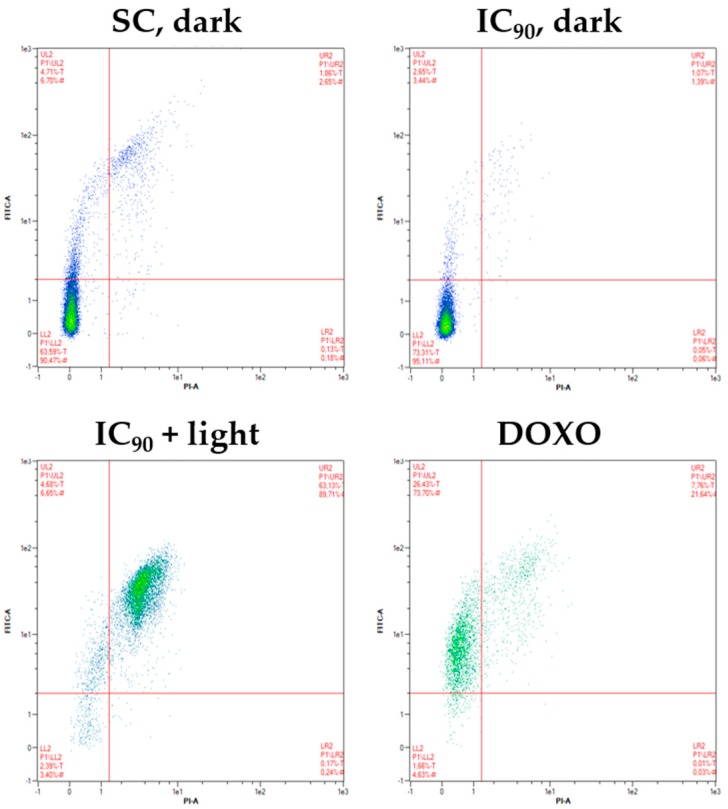
Representative dot plots after flow cytometric analysis of apoptosis induction in SISO cells. Cells were treated with solvent, DOXO, or the IC_90_ of mTHPC. Afterwards, cells were kept in the dark or illuminated with 1.8 J/cm^2^ and stained with Annexin V-FITC/PI 24 h after illumination. For each sample, 10,000 events were counted and gated for the single cell population (not shown). Unstained, vital cells appear in the lower left quadrant, Annexin V-FITC (apoptotic) cells in the upper left quadrant and Annexin V-FITC- and PI-positive (late-apoptotic) cells appear in the upper right part. The FITC channel (λ_Ex/Em_ = 488/525–550 nm) of a MACS Quant flow cytometer was used for the detection of Annexin V-positive cells and the PI channel (λ_Ex/Em_ = 488/655–730 nm) was used for the PI signal. Data were analyzed with the MACS Quantify Software.

Apoptosis has been shown to be an important route of cellular death involved after photodynamic therapy [26,27]. For the detection of apoptotic cells after mTHPC-based PDT, a flow cytometric analysis was used to visualize cells double-stained with Annexin V-FITC and propidium iodide (PI) (see Figure A3 in Appendix A for representative analysis data). With the known anticancer drug doxorubicin (DOXO), apoptotic cells were detected at all time points (Figure 6A–E). Compared to a solvent-treated, non-illuminated control, no increases of Annexin V-FITC single stained (apoptotic) or Annexin V-FITC/PI double-stained (late-apoptotic) cells were observed for solvent-treated and illuminated as well as mTHPC-treated, but non-illuminated controls at any time point.

For A-427, the IC_90_ in combination with light led to significantly more apoptotic cells compared to the solvent-treated dark control independently of the incubation time. After 6 h, 28.3%, and after 24 h, 37.6% of the cells were Annexin V-FITC-positive, whereas this fraction dropped to 7.9% after 48 h. However, it is noteworthy that at this time point the fraction of late-apoptotic cells reached its peak at 55.1%. A similar pattern was observed after mTHPC-based PDT applied to BHY cells. The amount of apoptotic cells increased over time for the IC_90_ from 13.8% (6 h) to 41.5% (48 h). Additionally, the IC_50_ led to more apoptotic cells (33.3%) 48 h after illumination. Late-apoptotic cells were significantly increased after 6 h (15.3%, IC_90_) and 48 h (19.7%, IC_50_ and 36.2%, IC_90_). RT-4 cells responded to mTHPC-based PDT at an early time point of 6 h with an increase of apoptotic cells (33.8%, IC_90_) as well as after 48 h (26.6%, IC_90_). In contrast to that, values after treatment with the IC_50_ and light gradually rose to peak 48 h after PDT at 28.8%. Late-apoptotic fraction was significantly increased only after 24 h (26.1%, IC_90_) and dropped after 48 h (9.1%). For KYSE-70 and SISO cells, similar results were detected by the flow cytometric analysis. For KYSE-70 cells, a slight increase of apoptotic cells was detected 6 and 48 h after treatment with the IC_90_ and for the former also with the IC_50_. For SISO cells, no significant increase of apoptotic cells was observed at any time point. Instead, the two cell lines responded to mTHPC-PDT with an early increase of the Annexin V-FITC- and PI-positive fraction after 6 h with 17.2% for KYSE-70 and 11.1% for SISO cells. After 24 and 48 h, both cells displayed similarly high levels of 37.5 and 43.9% (KYSE-70) as well as 55.2 and 48.7% (SISO), respectively.

#### 2.3.5. PARP Cleavage Confirms Induction of Apoptosis after mTHPC-PDT

**Figure 7:**
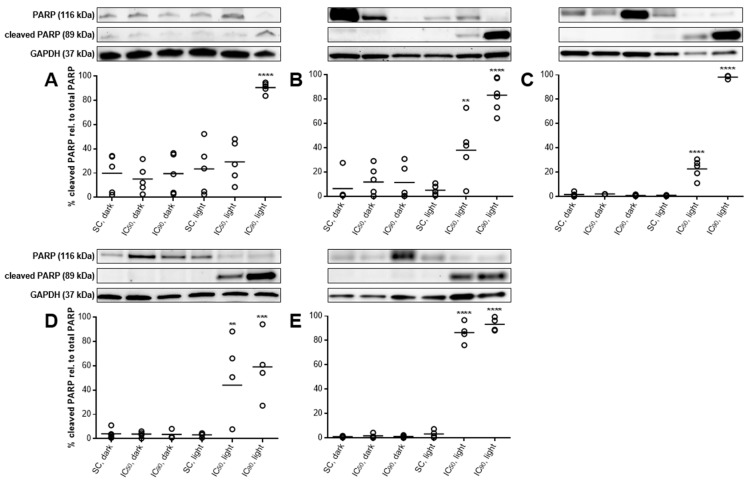
Poly(ADP-ribose) polymerase (PARP) cleavage in A-427 (**A**), BHY (**B**), KYSE-70 (**C**), RT-4 (**D**), and SISO (**E**) cells after mTHPC-PDT. Cells were treated with either solvent (SC) or equitoxic concentrations (IC_50_ or IC_90_ in Table 1) of mTHPC between 0.02–0.3 µM, illuminated with 1.8 J/cm^2^ or left in the dark, and total protein extracts were harvested 24 h after illumination and western blotting performed. Representative blots are shown and the % cleaved PARP relative to PARP was evaluated by densitometric analysis. GAPDH was used as a loading control. Data presented as dot plots from at least three independent experiments. Solvent-treated, non-illuminated cells served as the reference group. (** *p* < 0.01; *** *p* < 0.001; **** *p* < 0.0001).

The induction of apoptosis was also investigated by western blot analysis of PARP and its cleaved form, which is involved in the process of apoptosis [28]. PARP cleavage was observed in all tested cell lines, but not under all conditions (Figure 7A–E). In A-427, 90.6% of PARP were found in the cleaved form after treatment with the IC_90_ of mTHPC and illumination. Compared to the other cell lines, A-427 showed the highest amounts of cleaved PARP in the reference samples. In the other cell lines, the percentage of cleaved PARP relative to PARP was significantly increased to 38.1–86.4% (IC_50_) and 59.1–98.3% (IC_90_), respectively.

#### 2.3.6. PARP Cleavage at Least Partly Traced Back to Caspase 3-Activation

**Figure 8:**
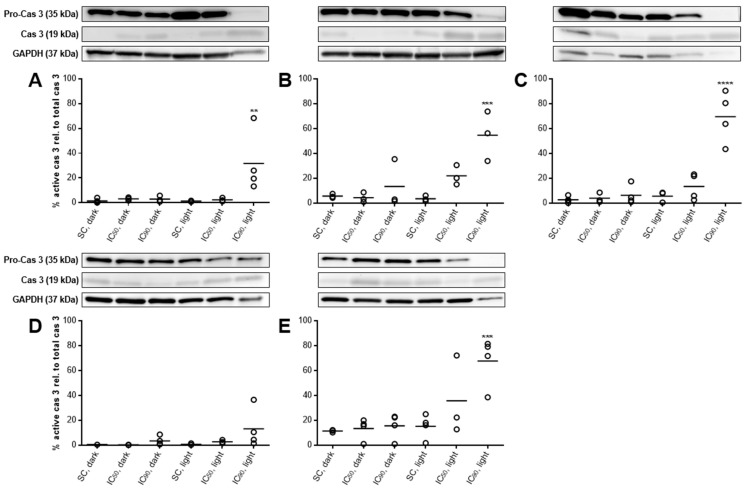
Caspase 3-activation in A-427 (**A**), BHY (**B**), KYSE-70 (**C**), RT-4 (**D**), and SISO (**E**) cells after photodynamic treatment. Cells were treated with either solvent (SC) or equitoxic concentrations (IC_50_ or IC_90_ in Table 1) of mTHPC between 0.02–0.3 µM, illuminated with 1.8 J/cm^2^ or left in the dark, and total protein extracts were harvested 24 h after illumination and western blotting performed. Representative blots are shown and % cas 3 relative to pro-cas 3 was evaluated by densitometric analysis. GAPDH was used as a loading control. Data presented as dot plots from at least three independent experiments. Solvent-treated, non-illuminated cells served as the reference group. (^**^ *p* < 0.01; ^***^ *p* < 0.001; ^****^ *p* < 0.0001).

Among others, PARP is one of the main cleavage targets of the active effector caspase 3. Therefore, the activation of caspase 3, e.g., via initiator caspases 8 (extrinsic) or 9 (intrinsic), is another common hallmark of apoptotic cell death [29], and was investigated by western blotting. Solvent-treated as well as mTHPC treated, but non-illuminated cells only displayed caspase 3 in its active form in low levels (Figure 8A–E). In general, SISO cells showed a higher background activation of caspase 3 in controls.

Activation of (pro-)caspase 3 was detected in four out of five tested cell lines after mTHPC-PDT, but the fraction of active caspase 3 relative to inactive pro-caspase 3 was significantly increased only after treatment with the IC_90_. While caspase 3 activation could only be detected to a low extent in RT-4 cells (13.3%) with this concentration, 31.8–69.7% of active caspase 3 were found in the other cell lines. The IC_50_ of mTHPC initiated caspase 3-activation only in BHY, KYSE-70, and SISO cells (13.5–35.9%), but no significant differences to solvent-treated cells were observed.

### 2.4. mTHPC-PDT Induces G_2_/M Arrest and the Formation of Sub G_1_ Populations with Fragmented DNA, Emphasizing the Induction of Apoptosis

**Figure 9:**
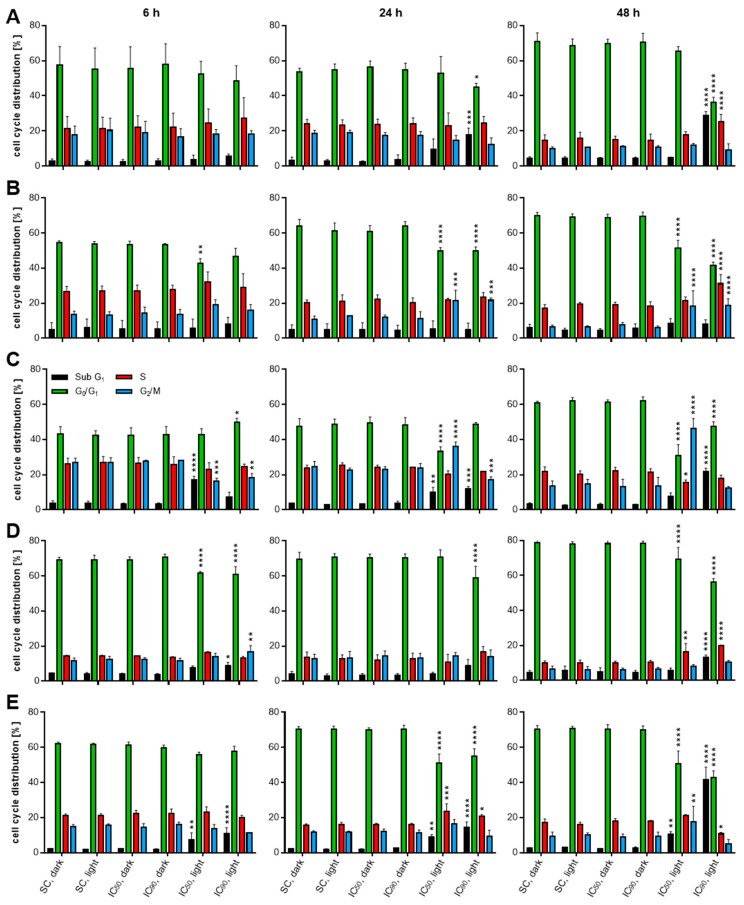
Cell cycle analyses of A-427 (**A**), BHY (**B**), KYSE-70 (**C**), RT-4 (**D**), and SISO (**E**) cells after mTHPC-PDT. Cells were treated with either solvent (SC) or equitoxic concentrations (IC_50_ or IC_90_ in Table 1 of mTHPC between 0.02–0.3 µM, illuminated with 1.8 J/cm^2^ or left in the dark, stained with PI and analyzed 6 (left), 24 (middle), or 48 h (right) after photodynamic treatment. Flow cytometric analysis of the single cell population was carried out using the PI channel (λ_Ex/Em_ = 488/655–730 nm). Cells were assigned to either sub G_1_ (black, fragmented DNA, apoptotic), G_0_/G_1_ (green), S (red), or G_2_/M (blue) phase. Data presented as means ± SD from at least three independent experiments. Solvent-treated, non-illuminated cells served as the reference group. (* *p* < 0.05; ** *p* < 0.01; *** *p* < 0.001; **** *p* < 0.0001).

**Figure A4:**
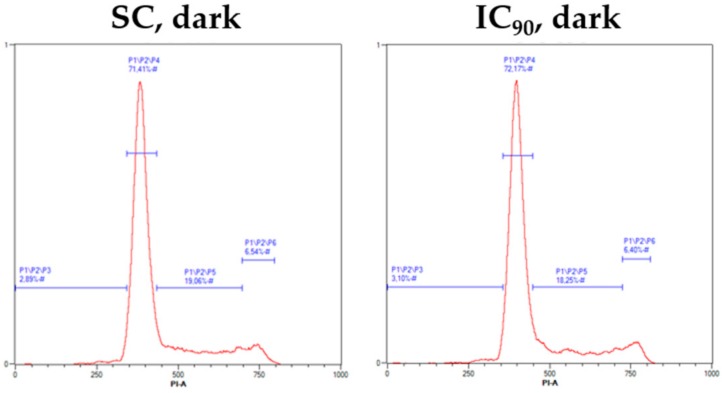

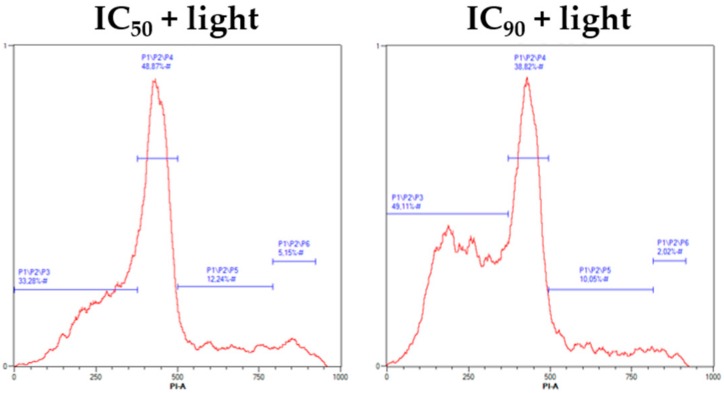
Representative histograms after flow cytometric analysis of cell cycle distribution in SISO cells. Cells were treated with solvent, and the IC_50_ or IC_90_ of mTHPC. Afterwards, cells were kept in the dark or illuminated with 1.8 J/cm^2^. Fixation and PI staining were carried out 48 h after illumination. For each sample, 10,000 events were counted and gated for the single cell population (not shown) and cells were assigned either sub G_1_ (fragmented DNA, apoptotic), G_0_/G_1_, S, or G_2_/M phase. The PI channel (λ_Ex/Em_ = 488/655–730 nm) of a MACS Quant flow cytometer was used for the detection of PI fluorescence. Data were analyzed with the MACS Quantify Software.

To further investigate whether the cell death mechanism is accompanied by growth inhibition or DNA fragmentation, which is another hallmark during apoptosis [30], cell cycle analysis was carried out after staining with PI. The analysis allowed for the assignment of cells in either sub G1 (fragmented DNA, apoptosis), G_0_/G_1_, S, or G_2_/m phase of the cell cycle (see Figure A4 in Appendix A for representative analysis data). The effects on the cell cycle were examined 6, 24, and 48 h after mTHPC-based PDT. Again, no effects on cell cycle distribution were observed after treatment with mTHPC in the absence of light or after illumination of solvent-treated cells for any cell line at any time point compared to solvent-treated cells incubated in the dark (Figure 9A–E).

For A-427 cells, an increase in sub G_1_ cells was detected 24 h after mTHPC-PDT leading to 9.6% (IC_50_) and 17.8% (IC_90_) of apoptotic cells, respectively. Similar results were obtained for RT-4 and SISO cells. For the RT-4 cell line, sub G_1_ population was significantly increased 6 h (8.9%) and 48 h (13.4%) after PDT with the IC_90_ of mTHPC. SISO cells displayed significantly more cells in the sub G_1_ phase for both concentrations after mTHPC-PDT at any tested time point (7.2%, 8.9% and 10.6% (IC_50_) and 10.8%, 14.4%, and 41.5% (IC_90_) after 6, 24, and 48 h). In addition, significantly more SISO cells (17.8%) showed an arrest in the G_2_/M phase 48 h after mTHPC-PDT. Diverging results were obtained for BHY and KYSE-70 cells. BHY cells showed no increase in sub G_1_ apoptotic cells at any time point, but G_2_/M population was significantly higher 24 h (21.4% and 21.7%) and 48 h (18.5% and 18.8%) after photodynamic treatment with the IC_50_ and IC_90_, respectively. For KYSE-70 cells, both an increased sub G_1_ population as well as G_2_/M arrest were observed. For the IC_50_, sub G_1_ cells were most dominant 6 h after PDT (17.2%) and dropped after 24 h (10.2%) and 48 h (7.6%). The opposite trend was observed for the IC_90_, where the sub G_1_ population increased over time (7.3%, 11.9%, and 21.8% after 6, 24, and 48 h) after mTHPC-PDT.

Additionally, KYSE-70 cells showed a significant time-dependent increase in the G_2_/M population after treatment with the IC_50_ (16.3%, 35.9%, and 46.2% after 6, 24, and 48 h), whereas a decrease in this population with time was observed for the IC_90_ (18.1%, 17.2%, and 12.3%). For all five cell lines, changes in S phase distribution played only a minor role and changes in G_0_/G_1_ population were consequences of increased or decreased sub G_1_ and G_2_/M populations.

### 2.5. Autophagic Flux Analysis by LC3-II Levels in the Absence and Presence of Lysosomal Protease Inhibitors Revealed a Simultaneous Occurrence of Autophagy and Apoptosis after mTHPC-PDT

**Figure 10:**
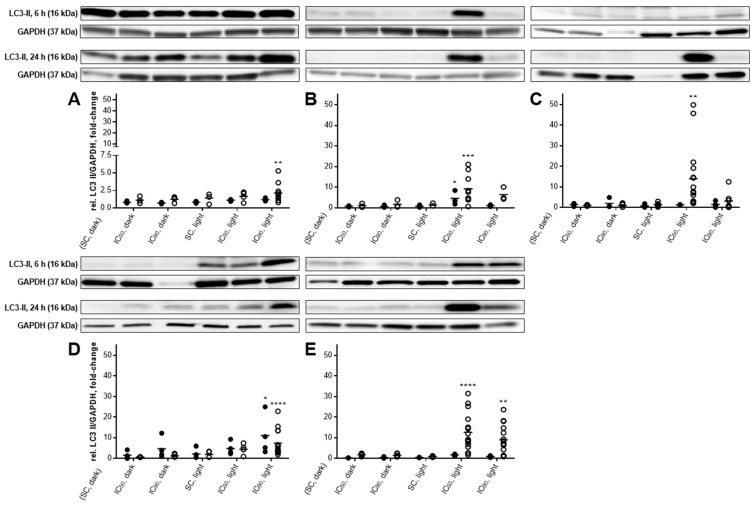
Formation of LC3-II in A-427 (A), BHY (B), KYSE-70 (C), RT-4 (D) and SISO (E) cells after photodynamic treatment. Cells were treated with either solvent (SC) or equitoxic concentrations (IC_50_ or IC_90_ in Table 1) of mTHPC between 0.02–0.3 µM, illuminated with 1.8 J/cm^2^ or left in the dark, and total protein extracts were harvested 6 (●) or 24 h (○) after illumination and western blotting performed. Representative blots are shown and the amount of LC3-II relative to a solvent-treated, non-illuminated reference control (LC3-II level for this sample was set to 1.0) was evaluated by densitometric analysis. GAPDH was used for normalization. Data presented as dot plots from at least three independent experiments. (** *p* < 0.01; *** *p* < 0.001; **** *p* < 0.0001).

**Figure 11:**
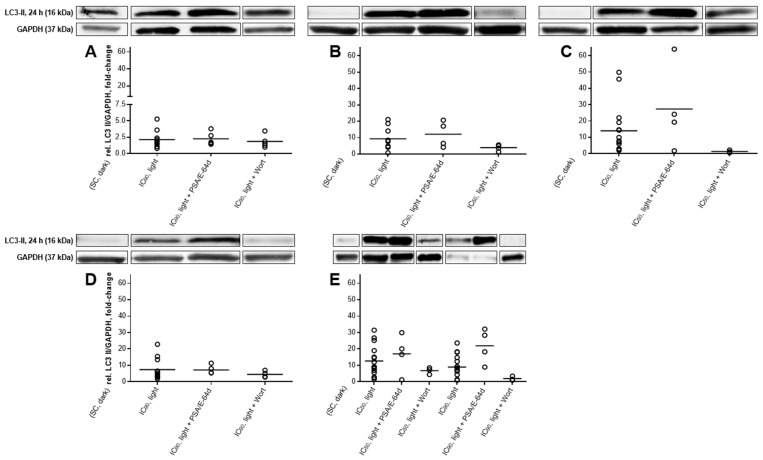
Formation of LC3-II in A-427 (**A**), BHY (**B**), KYSE-70 (**C**), RT-4 (**D**), and SISO (**E**) cells after photodynamic treatment in absence or presence of lysosomal protease inhibitors pepstatin A (PSA) and E-64d for autophagic flux detection or PI3K inhibitor wortmannin (Wort) for blocking of autophagic sequestration, respectively. Experiments were carried out with selected mTHPC concentrations that led to an increase of LC3-II levels (Figure 10). Cells were treated with equitoxic concentrations (IC_50_ or IC_90_ in Table 1) of mTHPC that led to elevated LC3-II levels in the absence of the particular inhibitor. Only selected concentrations were tested for inhibitor pretreatment was carried out with 100 µM PSA and 10 µg/mL E-64d for 4 h or with 2 µM wortmannin for 1 h before PDT and cells were then illuminated with 1.8 J/cm^2^ or left in the dark. Total protein extracts were harvested 24 h after illumination and western blotting performed. Representative blots are shown and the amount of LC3-II was evaluated by densitometric analysis. GAPDH was used for normalization. Data presented as dot plots from at least three independent experiments.

The involvement of autophagy in the fate of mTHPC-treated and illuminated cells was investigated by western blotting of LC3-II 6 and 24 h after PDT (Figure 10A–E). LC3-II levels in the absence of lysosomal inhibitors only reflect the formation of autophagosomes without any information about the overall autophagic flux. Therefore, lysosomal degradation of LC3-II was inhibited by treatment with pepstatin A and E-64d and LC3-II levels compared to results without lysosomal protease inhibitors [31,32]. Furthermore, the PI3K inhibitor wortmannin was used to block autophagic sequestration and therefore inhibit autophagy (Figure 11A–E) [31,33].

In the absence of lysosomal inhibitors, LC3-II levels were increased 6 h after PDT in BHY (4.6-fold, IC_50_) and RT-4 (11.0-fold, IC_90_) cells compared to a solvent-treated, non-illuminated control. The LC3-II levels of these reference samples were normalized to 1.0. After 24 h, treatment with the IC_50_ of mTHPC in combination with light slightly elevated LC3-II levels in BHY (9.3-fold), KYSE-70 (14.0-fold), and SISO (12.6-fold) cells, whereas RT-4 cells were less affected (4.5-fold) and A-427 cells remained unaffected. However, treatment with the IC_90_ led to a significant increase also in RT-4 (7.4-fold) and A-427 cells (2.1-fold). This was also true for SISO cells (9.0-fold), indicating an involvement of autophagy in cellular death irrespective of the concentration, at least for this cell line. Interestingly, and unlike the IC_50_, the higher mTHPC concentration did not have a significant effect on LC3-II levels in BHY and KYSE-70 cells.

Autophagic flux was investigated for each cell line only 24 h after PDT with mTHPC concentrations that led to increased levels of LC3-II in the absence of lysosomal protease inhibitors. After pretreatment with pepstatin A and E-64d, higher levels of LC3-II were observed in BHY (12.1-fold, IC_50_), KYSE-70 (27.3-fold, IC_50_), and SISO cells (17.0-fold, IC_50_ and 22.0-fold, IC_90_) 24 h after mTHPC-PDT than in the absence of the inhibitors (Figure 11A,D). However, no change in the levels of LC3-II were detected in A-427 and RT-4 cells after exposure to lysosomal protease inhibitors.

Likewise, blockage of autophagy induction by wortmannin was carried out for each cell line 24 h after PDT at mTHPC concentrations that led to increased levels of LC3-II in the absence of the PI3K inhibitor. Lower LC3-II levels were detected in all cell lines 24 h after mTHPC-based PDT and wortmannin pretreatment (Figure 11A–E). More precisely, LC3-II formation was still increased in A-427 (1.9-fold, IC_90_), BHY (3.9-fold, IC_50_), KYSE-70 (1.3-fold, IC_50_), RT-4 (4.5-fold, IC_90_), and SISO cells (6.7-fold, IC_50_ and 2.0-fold, IC_90_) in comparison to the solvent-treated, non-illuminated controls, but was substantially lower than for the same treatment without wortmannin.

## 3. Discussion

The analysis of cellular viability after PDT with a light dose of 1.8 J/cm^2^ by the MTT assay revealed that mTHPC-PDT was an effective photosensitizer against all five cell lines from different tissue origins (Figure 1). Notably, large differences were observed in susceptibility towards the photosensitizer among the cell lines, with A-427 lung cells being most and RT-4 bladder cells being the least sensitive cell line. Dark toxicity of mTHPC was observed only with high concentrations and the IC_50_ values were 17–30 times greater than the respective value after light incubation. These results indicate that mTHPC is non-toxic in the absence of light, at least within a PDT-relevant concentration range. The data confirmed the effectiveness of mTHPC-PDT in substantially lowering cellular viability of human tumor cells. However, the administered mTHPC concentration has to be adjusted to the target tissue to ensure a high efficacy in tumor elimination. In comparison to published data, similar IC_50_ values (0.02–0.1 µM) were observed with mTHPC in the MTT assay by other groups 24 h after PDT with a comparable light dose of 1.5 J/cm^2^. Berlanda and colleagues determined an IC_50_ of 0.03 µM in the epidermoid carcinoma cell line A-431 [34] and Kiesslich et al. established IC_50_ values of 0.07 µM in a human bile duct (BDC) and a gall bladder cell line (GBC) [17]. However, higher IC_50_ values of 1.6 µM were determined by Abdulrehman et al. in colon cell lines SW480 and SW620 [35].

**Figure A5:**
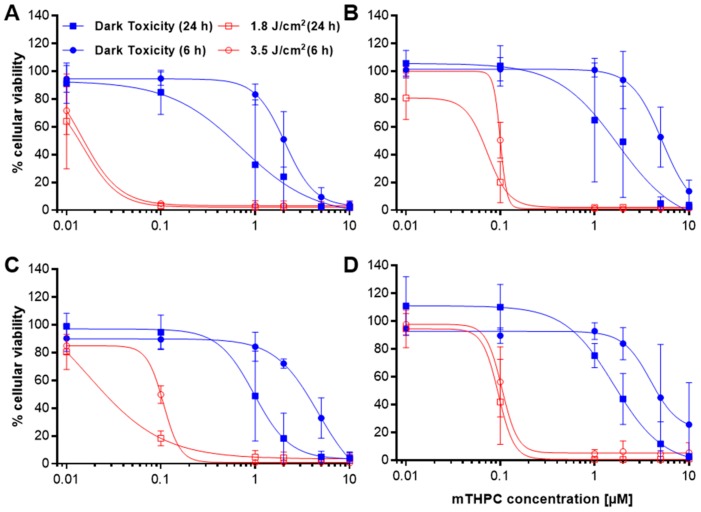

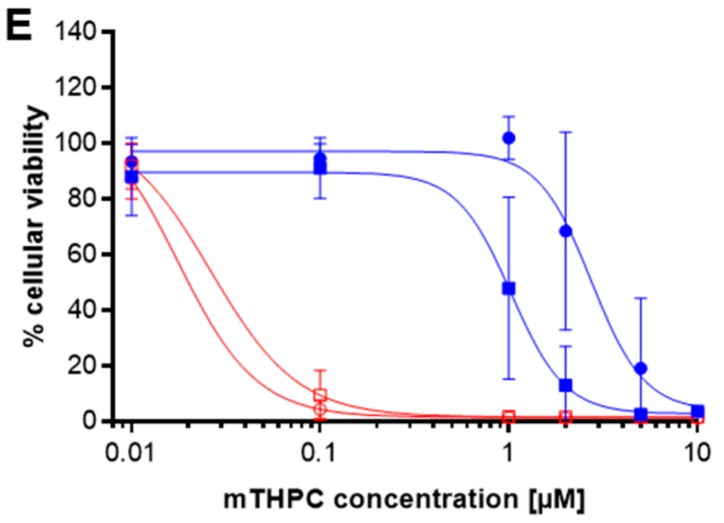
Dark (●, 6 h; ■, 24 h) and light-induced (○, 6 h; □, 24 h) loss of cellular viability after mTHPC treatment as assessed by the MTT viability assay (with remaining cells from LDH release assay). The five cell lines A-427 (**A**), BHY (**B**), KYSE-70 (**C**), RT-4 (**D**), and SISO (**E**) were treated with mTHPC for 24 h in concentrations ranging from 0.01–10.0 µM and kept in the dark (dark toxicity) or illuminated with a light dose of 1.8 J/cm^2^ or 3.5 J/cm^2^ (light-induced toxicity). MTT assay was carried out 6 h or 24 h post illumination and the absorbance of the reduced formazan was measured at λ = 570 nm. The percentage of cell viability was calculated by dividing the absorbance for the treated group by the absorbance in the solvent dark control. Results presented as means ± SD from at least three independent experiments.

The determination of cellular membrane integrity after mTHPC-PDT was assessed by LDH release into the supernatant (Figure 2). In the dark, a dose-dependent increase in cytotoxicity was observed at concentrations starting with 1.0 µM or higher. After illumination, no substantially increased cytotoxicity was detected except for A-427, RT-4, and SISO cells. However, these differences were only seen at medium concentrations, while at higher concentrations LDH release surprisingly decreased to levels comparable to those observed without illumination. These results indicate that necrosis is only induced in some cell lines at rather low concentrations and does not contribute to cellular death after application of high mTHPC concentrations. These findings were confirmed by a subsequent MTT cell viability assay that has been carried out with the cells that remained in the 96-well plates after removal of the supernatant used for the LDH release assay (see Figure A5 in Appendix A). The MTT data revealed that cellular viability dropped to <5% in the absence of light at mTHPC concentrations of 5 µM (24 h) and 10 µM (6 h; <15% for BHY and <30% for RT-4 cells). More interestingly, after illumination with either 3.5 or 1.8 J/cm^2^, cellular viability was decreased to <5% at mTHPC concentrations of 0.1–0.3 µM (1.0 µM for KYSE-70), which implies that cellular membrane integrity was still intact, although cellular viability was nearly completely lost under these conditions. The presence of an intact cellular membrane accompanied by a nearly total loss of cellular viability clearly indicates that little or no necrosis occurred during cellular death. In the case of necrosis, a total loss of cellular viability would have led to high levels of LDH released into the supernatant due to a porous membrane. Treatment with Triton X-100 led to minimal residual cellular viability in the MTT assay (see Table A1 in Appendix A).

Altogether, these results indicate that the induction of necrosis plays only a minor role after mTHPC-PDT in the tested cell lines. Similar findings were reported by Löw et al. after the treatment of colon carcinoma cell line HT29 with mTHPC in similar concentrations of 0.15–15 µM and an even higher light dose of 5.0 J/cm^2^ [36]. In the LDH release assay, that group found that the relative cytotoxicity reached values of >15% in the absence of light at mTHPC concentrations of 7.5 and 4.5 µM after incubation times of 4 and 24 h, respectively. These results are consistent with our results obtained with the five tested cell lines (except KYSE-70 cells). After illumination of the cells, Löw et al. detected no substantial cytotoxicity 4 h after PDT and similar levels of cytotoxicity 24 h after PDT compared to the values observed after incubation in the dark. These results were again consistent with those we found with all five tested cell lines. Additionally, Löw and colleagues used the WST-1 assay and detected a total loss of cellular viability at 7.5 and 1.5 µM after illumination with 5.0 J/cm^2^ followed by an incubation period of 4 and 24 h, respectively. These results are again consistent with our results obtained with the MTT assay, where a total loss of cellular viability was observed 6 or 24 h after illumination with 3.5 J/cm^2^ for mTHPC concentrations of 0.1–1.0 µM.

ROS-induced toxicity is one characteristic outcome of PDT [37]. Furthermore, membrane lipids are known targets for oxidation during oxidative stress [38]. In our study, ROS could be detected in KYSE-70 and SISO cells after illumination with 1.8 J/cm^2^, but higher light doses were required for A-427 and BHY cells (Figure 3). However, substantial loss of cellular viability was observed with doses of ≤1.8 J/cm^2^ in all five cell lines. At time points 24 and 48 h after mTHPC-PDT, lipid peroxidation (LPO) was additionally detected in KYSE-70, RT-4 and SISO cells (Figure 4). Surprisingly, enhancement of LPO occurred in RT-4 cells, although no increased ROS levels were observed after illumination with 1.8 J/cm^2^. One explanation could be the limitation of H_2_DCF-DA to detect singlet oxygen (^1^O_2_) [19]. Indeed, ^1^O_2_ could even act as a quencher of DCF fluorescence [39]. The singlet oxygen quantum yield (*Φ*_∆_) of mTHPC ranges from 0.3–0.4 in different solvents [40,41], meaning that 30–40% of the energy of each photon absorbed by mTHPC can be used for the generation of ^1^O_2_ from triplet oxygen (^3^O_2_). The remaining energy can be transferred for the generation of other ROS, e.g., superoxide anion (O_2_^●−^) or hydroxyl radicals (OH^●^), or will instead be lost via fluorescence emission or thermal radiation [1]. If the proportion of ^1^O_2_ on total ROS was in the same range of 30–40% in our ROS measurements, it may led to increases of ROS that were not detectable by the DCF assay, while detectable species like O_2_^●−^ or OH^●^ did not increase substantially. High but undetectable levels of ^1^O_2_ could led to the increase of LPO in KYSE-70, RT-4 and SISO cells after illumination with 1.8 J/cm^2^, although no increase in ROS levels were observed under these conditions by H_2_DCF-DA staining. A further explanation for not observing an increase in ROS levels could be a high efficacy of anti-oxidative pathways, e.g., due to high levels of catalase, glutathione peroxidase (GP_X_), glutathione (GSH), or GSH-recovering enzymes like glutathione-disulfide reductase (GSR). For example, relatively high levels of GSH have been found for RT-4 cells by our group, while the other cell lines displayed lower GSH concentrations [42]. However, the generated ROS would still have to exist long enough to cause oxidative stress and induce a loss of cellular viability. No enhanced LPO as well as no increased ROS levels were observed in A-427 and BHY cells. Furthermore, no substantial loss of membrane integrity has been detected in the LDH release assay in any cell line after mTHPC-mediated PDT (Figure 2), suggesting that the cellular membrane is not the primary target of mTHPC-PDT. These results indicate that LPO plays only a minor role in photodamage after mTHPC-PDT (at least in A-427 and BHY cells) and is not correlated with ROS levels and cellular viability.

The role of LPO in cellular death after PDT has been subject of discussion in the past. Ehrenberg and colleagues concluded that the toxicity after PDT with hematoporphyrin (HP) resulted from damage of proteins rather than from LPO-induced membrane damage [43]. Gaullier and co-workers also detected no correlation between LPO and cellular death after PDT with protoporphyrin IX (PpIX) [44]. With mTHPC as the applied PS, Klein et al. detected enhanced LPO and oxidation of proteins after treatment with mTHPC (5–50 µg/mg mitochondrial protein) and illumination with a high light dose of 5.3 J/cm^2^. However, experiments were carried out with isolated rat liver mitochondria and therefore do not reflect the situation in whole cells properly [45]. Melnikova and co-workers treated HT29 colon adenocarcinoma cells with 1.5 µM mTHPC and light doses of 2.3–6.8 J/cm^2^ and concluded that LPO only plays a minor role in cell inactivation by mTHPC-PDT [46]. In a study of Kirveliene and colleagues with two rodent cell lines, cells were treated with 0.75 µM mTHPC and illuminated with a light dose of 1.8 J/cm^2^. Enhanced LPO was found as an early response to mTHPC-PDT, but the cells were able to restore LPO to initial levels already 2 h after light exposure. Additionally, the group found neither LDH nor ATP release after mTHPC-PDT. These findings were consistent with our results from the LDH release assay (Figure A5 in Appendix A), suggesting that a loss of membrane integrity by LPO and induction of necrosis do not occur [47].

The induction of oxidative stress triggers apoptosis accompanied or preceded by a loss of mitochondrial membrane potential (Δ*ψ*_m_) [24,25]. In our study, a collapse of Δ*ψ*_m_ was observed after mTHPC-mediated PDT in all of the five tested cell lines (Figure 5). These results indicate a direct targeting of the mitochondria by mTHPC-PDT and prove the induction of apoptosis at an early stage. These findings are consistent with results from Marchal and colleagues with the JC-1 dye in flow cytometric approaches. Depolarization was observed 4 and 24 h after PDT in HT29 human adenocarcinoma cells after treatment with 1.5–4.5 µM mTHPC and light doses of 0.06–1.9 J/cm^2^ [48,49]. Treatment of human breast adenocarcinoma cell line MCF-7 with 1.5 µM mTHPC also led to a loss of Δ*ψ*_m_ immediately and 24 h after illumination. In this study, the group also proved that mTHPC induced direct mitochondrial photodamage rather than an indirect damage via the translocation of the pro-apoptotic Bax protein [16].

The induction of apoptosis was investigated 6, 24 and 48 h after illumination with a light dose of 1.8 J/cm^2^ by staining with Annexin V-FITC and PI (Figure 6). It has to be mentioned that Annexin V-FITC/PI double-stained cells need to be considered as late-apoptotic and not necrotic one as the LDH release assay revealed no signs of necrosis involvement in cellular death. Misinterpretation of late-apoptotic as necrotic cells has been an issue in the past after mTHPC-PDT, where further confirmation of necrosis has not been carried out [48,49]. In all tested cell lines, a time-dependent increase in apoptotic (A-427, BHY) or late-apoptotic (A-427, KYSE-70, RT-4, SISO) populations can be observed for the high mTHPC concentration, indicating that even high-dose PDT leads to apoptosis rather than necrosis. Furthermore, a switch from apoptosis to late apoptosis was observed over time. Apoptosis induction after mTHPC-PDT was also observed via Annexin V-FITC/PI staining by Marchal and colleagues in HT29 human adenocarcinoma cells (1.5 µM, 1.92 J/cm^2^) [49] and Abdulreham et al., who found apoptotic populations in colon carcinoma cell lines SW480 and to a lesser extent in SW620 with no evidence of late-apoptotic or necrotic cells (0.2–11.8 µM, 6.0 J/cm^2^) [35]. Furthermore, Yow and colleagues visualized phosphatidylserine externalization in the human nasopharyngeal carcinoma cell line NPC/HK1 by confocal laser scanning microscopy (1.2 µM, 2.0 J/cm^2^) [50].

Apoptosis induction was further studied and confirmed by the cleavage of PARP and the activation of caspase 3 (Figure 7 and Figure 8). Our findings support the results observed by the Annexin V-FITC/PI method. However, the IC_50_ led to PARP cleavage to a lesser extent and it should be emphasized that no or only little signs of apoptosis could be detected for this concentration, indicating that PARP might be additionally cleaved via a different pathway. Caspase 3-activation and PARP cleavage were observed after mTHPC-PDT under comparable conditions by other groups, e.g., in human breast carcinoma cell line MCF-7 (1.5 µM, 0.01–0.06 J/cm^2^) [16] and HT29 adenocarcinoma cells (1.5–4.5 µM, 0.3–1.9 J/cm^2^) [48,49]. The detection of a 89 kDa proteolytic fragment of cleaved PARP is characteristic after activation of the effector caspase 3, since PARP is a substrate of active caspase 3 [29]. In our study, caspase 3-activation was consistent with the results that were obtained by Annexin V-FITC/PI staining. Caspase 3 is directly connected to phosphatidylserine externalization via the inhibition of flippase and the activation of scramblase during apoptosis, two enzymes involved in the maintenance of membrane asymmetry in vital cells [51,52,53]. While caspase 3-activation was followed by PARP cleavage for the IC_90_ concentration, it was not the case for the IC_50_ concentration. These findings further support the hypothesis that PARP cleavage was also initiated by an additional route, e.g., by active caspase 7, which also produces a 89 kDa proteolytic PARP fragment [54,55,56]. Furthermore, the involvement of PARP as a suppressor in autophagy may also lead to an additional cleavage after oxidative stress [57,58]. Caspase-dependent PARP cleavage also circumvents necrotic cell death by preventing the depletion of NAD^+^ and ATP by overactivated PARP [55,59,60], further indicating that necrosis plays only a minor role in mTHPC-PDT. It is a generally accepted statement that PDT leads to apoptosis with low and medium PS concentrations and rather necrosis with high PS concentrations in several cell types [61,62], which has also been shown with mTHPC as the selected PS [13,48,63]. However, our data do not point at necrosis as a cause of cell death for mTHPC-PDT.

Effects on the cell cycle were observed after mTHPC-PDT (Figure 9). Fragmented DNA can be detected in cells of the sub G_1_ fraction and is characteristic for the final steps of apoptosis downstream of caspase 3/7-activation and PARP cleavage. Namely, caspase-activated DNase (CAD) is responsible for the degradation of DNA in the nucleus, but also caspase-independent pathways can be involved, e.g., after release of apoptosis-inducing factor (AIF) or the DNase EndoG from mitochondria [30,64,65]. In our studies, DNA fragmentation was observed and was in some cases accompanied by a G_2_/M arrest. However, BHY cells displayed a G_2_/M-arrested population, but no DNA fragmentation. The observed results for DNA fragmentation were consistent with the directly related caspase 3-activation and PARP cleavage for A-427, KYSE-70 and SISO cells, but divergent results were obtained with the BHY and RT-4 cell lines. In BHY, caspase 3-activation and PARP cleavage were clearly observed for mTHPC-PDT. However, neither the caspase 3-related activation of CAD nor the suppressed PARP-related DNA excision repair and repair of single- and double-strand breaks led to increased DNA fragmentation. Interestingly, significant G_2_/M arrest and even increased DNA replication were observed, indicating that BHY cells may try to compensate DNA-related damages, e.g., via an increased frequency of alternative repair mechanisms like hyper-homologous recombination repair [66,67]. Significant PARP cleavage was also detected in RT-4 cells, which was not preceded by substantial caspase 3-activation. Furthermore, no DNA fragmentation was observed, which might be explained by a missing caspase 3-related CAD activation. Therefore, it seems likely that PARP cleavage was initiated via an alternative route, but—like in BHY cells—the absent DNA repair activity did not lead to an accumulation of fragmented DNA in RT-4 cells. Nevertheless, a prominent sub G_1_ population also indicated DNA fragmentation 48 h after PDT in RT-4 cells.

Autophagy was investigated via western blotting by detecting LC3-II, which can be correlated with autophagosome numbers (Figure 10) and was confirmed by additional incubation with the PI3K inhibitor wortmannin, which blocks autophagic sequestration (Figure 11). Elevated LC3-II levels have been detected in all cell lines following mTHPC-PDT. Incubation with wortmannin led to decreases in LC3-II levels, but not that clearly in A-427 cells. These results indicate that autophagy was induced after mTHPC in all tested cell lines except A-427 cells.

In the presence of PSA and E-64d, LC3-II levels were increased, and therefore autophagic flux detection confirmed the induction of autophagy in these cell lines (Figure 11). However, autophagic flux was not enhanced in in A-427 and RT-4 cells. One explanation could be a late stage suppression of autophagy, e.g., by blocking autophagosome maturation or autophagosome-lysosome fusion. Similar results were obtained also by Kukcinaviciute et al. 24 h after mTHPC-PDT with HCT116 colorectal carcinoma cells after treatment with 0.15 µM mTHPC and light doses of 0.9–2.7 J/cm^2^. The amount of autophagosomes was raised, but no autophagic flux was observed [68], which are consistent with the results of François and colleagues [15]. The increase in LC3-II levels coincides with phosphatidylserine externalization (Figure 6), caspase 3-activation (Figure 8), and PARP cleavage (Figure 7) in A-427 and RT-4 cells, indicating that autophagy contributes to cellular death. However, no autophagic flux could be detected in these cell lines, leading to the conclusion that autophagy was inhibited at a late stage. In this case, no autophagy-associated cell death would occur, but the loss of pro-survival effects of autophagy may contribute to a further progression of apoptosis. For the lower concentration, neither apoptosis nor autophagy were detected, but PARP cleavage was substantial in RT-4 cells. In BHY and KYSE-70 cells, LC3-II levels were mainly increased at the lower mTHPC concentration, where no phosphatidylserine externalization and caspase 3-activation were observed. However, PARP cleavage occurred at this concentration, which could be due to a cleavage by active caspase 7. These findings indicate, that autophagy was rather a survival strategy, which counteracts apoptosis. This survival strategy ultimately fails for the lower concentration, probably leading to autophagy-associated cell death. At the high mTHPC concentration, autophagy was not involved in cellular death of BHY and KYSE-70 cells. An ambiguous picture has been produced by SISO cells. While phosphatidylserine externalization and caspase 3-activation were observed only in small amounts for the IC_50_, both were prominent after PDT with the IC_90_. PARP cleavage and increased LC3-II levels were, however, found with both mTHPC concentrations and autophagy induction was confirmed by autophagic flux analysis. These findings indicate that autophagy was cytoprotective after PDT with a low mTHPC concentration, which was confirmed by a missing increase in apoptotic cells 48 h after PDT. But at the high mTHPC concentration, autophagy tends to accompany cellular death and failed to prevent apoptotic cell death. Especially after PDT with PS that target the ER and the mitochondria, like mTHPC does [69,70], so called autophagy-associated cell death was observed after PDT. In contrast, also pro-survival effects of autophagy after PDT can occur, especially at low-level PDT [15,19,61,62,71,72,73,74]. However, our results suggest that the role of autophagy even varies with the same PS in different cell lines.

## 4. Materials and Methods

### 4.1. Cell Culture

The five different human cancer cell lines A-427 (lung carcinoma; ACC 234), BHY (oral squamous cell carcinoma; ACC 404), KYSE-70 (esophageal squamous cell carcinoma; ACC 363), RT-4 (urinary bladder transitional cell carcinoma; ACC 412), and SISO (cervix adeno carcinoma; ACC 327) were obtained from Leibniz Institute DSMZ (Braunschweig, Germany) and routinely checked for mycoplasma. All lines were cultured in phenol red containing RPMI 1640 medium (PAN Biotech, Aidenbach, Germany) supplemented with 10% (*v*/*v*) fetal bovine serum (FBS; Sigma-Aldrich, Munich, Germany), 100 µg/mL streptomycin and 100 U/mL penicillin G (PAN Biotech, Aidenbach, Germany) at 37 °C and 5% CO_2_ in a humidified atmosphere. During and post illumination the cells were cultured in the same medium, but without phenol red. Cells were subcultured by detachment with 0.5 g trypsin/0.2 g EDTA (Sigma-Aldrich, Munich, Germany) once a week. Individual experiments were done by using either transparent, flat-bottom 96-well plates with cells seeded out at a density of 2.0–5.0 × 10^3^ cells per well in 100 µL medium for measurement of cellular viability and lactate dehydrogenase (LDH) release, T25 flasks with cells seeded out at a density of 5.0 × 10^5^ cells in 5 mL medium for flow cytometric analyses (except ROS detection), 6-well plates with cells seeded out at a density of 2.5 × 10^5^ cells per well in 2 mL medium for western blot analyses and detection of ROS generation, or 4-well cell culture chamber slides with cells seeded out at a density of 7.5 × 10^4^ cells per chamber in 1 mL medium for fluorescence microscopy (all culture dishes from Sarstedt, Nümbrecht, Germany). After seeding, cells were allowed to attach and grow for 24 h before treatment.

### 4.2. Photosensitizer Treatment

mTHPC was kindly supplied by Biolitec AG (Jena, Germany). A 20 mM stock solution was prepared in propylene glycol/ethanol (60:40) and stored at 4 °C in the dark. The PS was added to the cells in RPMI 1640 medium containing 10% (*v*/*v*) FBS and left for 24 h. For detection of cellular viability and LDH release, concentrations between 0.001–5.0 µM were used. However, for the determination of phosphatidylserine externalization, cell cycle distribution, mitochondrial membrane potential and western blot analyses of caspase 3 (cas 3), poly(ADP-ribose) polymerase (PARP), and microtubule-associated protein light chain 3 (LC3-II), equitoxic concentrations corresponding to the IC_50_ and IC_90_ values (concentrations, where 50% and 90% of the measured effect, i.e., loss of cellular viability, was observed) established from the MTT cell viability assay data 24 h after illumination with 1.8 J/cm^2^ were used for the treatment (see Section 2.1). Concentrations ranged between 0.02–0.3 µM depending on the cell line.

### 4.3. Photodynamic Treatment

The mTHPC-treated cells were washed with PBS and fresh phenol red free RPMI 1640 medium containing 10% (*v*/*v*) FBS was added before illumination. Cells were illuminated with an LED array based illumination device with 432 LEDs (light-emitting diodes) following the example of Pieslinger et al. [75]. The LEDs (Kingbright, Issum, Germany) produced a wavelength spectrum of λ = 640–660 nm and a light dose of 1.8 J/cm^2^, 3.5 J/cm^2^, and 7.0 J/cm^2^ was applied at fluence rates of 3.0 mW/cm^2^ and 5.8 mW/cm^2^, respectively. Cells were harvested by trypsinization 6, 24, or 48 h post illumination for analyses. For detection of ROS, cells were harvested immediately after illumination. For the determination of dark toxicity, cells were treated with mTHPC, but not illuminated. A sample treated with solvent in medium (equal to the highest solvent concentration used for dilution of mTHPC in the respective assay) and kept in the dark served as a reference control in the assays (solvent-treated control, SC).

### 4.4. MTT Cell Viability Assay

Cellular viability was measured 24 h after illumination of the cells by using the MTT (3-(4,5-dimethyl-2-thiazolyl)-2,5-diphenyl-2*H*-tetrazolium bromide; Alfa Aesar, Karlsruhe, Germany) assay. After photodynamic treatment, 20 µL of a 2.5 mg/mL MTT solution were added to each 100 µL medium per well and incubated at 37 °C and 5% CO_2_ in a humidified atmosphere for 4 h. Supernatant was replaced afterwards with 50 µL DMSO per well and the absorbance of the reduced formazan was measured at λ = 570 nm with a microplate reader (SpectraMax Plus 384; Molecular Devices, Biberach, Germany). The percentage of cell viability was calculated by dividing the absorbance in the treated group by the absorbance in the solvent control. Calculation of the IC_50_ values was done with the help of Prism 6 (GraphPad Software, La Jolla, CA, USA).

### 4.5. LDH Release Assay

Cells were illuminated with 1.8 J/cm^2^ and 3.5 J/cm^2^ in phenol red free medium containing 2.5% (*v*/*v*) FBS and incubated for 6 and 24 h post illumination, respectively. The loss of membrane integrity as a sign of necrosis was determined by measurement of released LDH into the medium supernatant as described elsewhere [18]. Briefly, after centrifugation of the 96-well plate at 2.000 rpm for 5 min, 50 µL/well of the supernatant were transferred to a new plate and 50 µL of LDH buffer was added (remaining cells were used for a subsequent MTT cell viability assay). LDH buffer consisted of 0.9 mM INT (2-(4-Iodophenyl)-3-(4-nitrophenyl)-5-phenyl-2*H*-tetrazolium chloride; Sigma-Aldrich, Munich, Germany), 0.4 mM PMS (phenazine methosulfate; Alfa Aesar, Karlsruhe, Germany), 1.8 mM NAD, and 74 mM lactic acid (both Carl Roth, Karlsruhe, Germany) in 0.2 M Tris buffer, pH 8.0. After incubation for 30 min in the dark, the enzyme reaction was stopped by the addition of 50 µL/well stop solution (1 M HCl) and the absorbance of the reduced INT equivalent was measured at λ = 490 nm with a SpectraMax Plus 384 microplate reader. Maximum LDH release was assessed by addition of 10 µL 10x lysis solution (9% (*v*/*v*) Triton X-100; Sigma-Aldrich, Munich, Germany) to the cells in 100 µL medium and served as the reference. As a positive control, cells were treated with 0.1% (*v*/*v*) and 0.01% (*v*/*v*) Triton X-100 for 6 and 24 h, respectively.

### 4.6. Flow Cytometry

#### 4.6.1. Analysis of ROS Generation

The generation of ROS was analyzed after treatment with 2′,7′-dichlorodihydrofluorescein diacetate (H_2_DCF-DA; Sigma-Aldrich, Munich, Germany) as described elsewhere [76]. Flow cytometric analysis of fluorescent DCF was carried out directly after illumination with 1.8 J/cm^2^, 3.5 J/cm^2^ or 7.0 J/cm^2^ with a MACS Quant flow cytometer (Miltenyi Biotech, Bergisch Gladbach, Germany). For each sample, 10,000 events were counted and gated for the single cell population. Fluorescent 2′,7′-dichlorofluorescein (DCF) is formed within the cells after contact with ROS and the detected fluorescence intensity is increased with higher amounts of ROS [20,21]. The FITC channel (λ_Ex/Em_ = 488 nm/525–550 nm) was used for the detection of DCF and data were analyzed with the MACS Quantify Software (Miltenyi Biotech, Bergisch Gladbach, Germany). As a positive control, cells were treated with 1.0–2.0 mM H_2_O_2_ (Sigma-Aldrich, Munich, Germany) for 10 min.

#### 4.6.2. Detection of Lipid Peroxidation (LPO)

The detection of lipid peroxidation (LPO) was carried out after staining with BODIPY^665/676^ (Thermo Fisher Scientific, Waltham, MA, USA). Briefly, after treatment with equitoxic concentrations corresponding to the IC_50_ and IC_90_ values determined in the MTT cell viability assay for mTHPC, more precisely with concentrations between 0.02–0.30 µM depending on the cell line, 5.0 × 10^5^ cells were harvested by trypsinization 6, 24, and 48 h after subsequent photodynamic treatment. Cells were washed twice with PBS and stained with a 4.0 µM BODIPY^665/676^ solution (in PBS) at room temperature for 30 min in the dark. Afterwards, cells were washed with PBS and analyzed with a MACS Quant flow cytometer. For each sample, 10,000 events were counted and gated for the single cell population. The APC channel (λ_Ex/Em_ = 635/655–730 nm) was used for the detection of fluorescent BODIPY^665/676^. After accumulation in the cellular membrane, the dye is oxidized after contact with hydroxyl (OH^•^), alkoxyl (RO^•^), and in particular peroxyl radicals (ROO^•^) [23]. After reaction with radicals, a change in the fluorescence spectrum of BODIPY^665/676^ occurs, that can be detected by flow cytometric analysis [22]. Data were analyzed with the MACS Quantify Software. As a positive control, cells were treated with 0.4–3.0 mM *tert*-butyl hydroperoxide (*t*-BHP; Sigma-Aldrich, Munich, Germany) for 24 h (A-427: 0.2–0.4 mM *t*-BHP for 4 h), a known trigger of lipid peroxidation [77].

#### 4.6.3. Phosphatidylserine Externalization

Phosphatidylserine externalization was detected with the Annexin V-FITC Kit (Miltenyi Biotech, Teterow, Germany) according to the kit instructions. Cells were treated and 5.0 x 10^5^ harvested 6, 24, and 48 h after photodynamic treatment as described for the detection of lipid peroxidation. Cells were washed with Binding Buffer and stained with Annexin V-FITC at room temperature for 15 min in the dark. Cells were washed again and propidium iodide (PI) was added immediately before flow cytometric detection with a MACS Quant flow cytometer. For each sample, 10,000 events were counted and gated for the single cell population. The FITC channel (λ_Ex/Em_ = 488/525–550 nm) was used for the detection of Annexin V-positive cells, PI-positive cells were detected with the PI channel (λ_Ex/Em_ = 488/655–730 nm). Data were analyzed with the MACS Quantify Software. As a positive control, cells were treated with 0.5–5.0 µM doxorubicin (DOXO; Pfizer, New York, NY, USA), a well-known inducer of ROS and apoptosis [78,79].

#### 4.6.4. Cell Cycle Analysis

Cell cycle was analyzed after staining with PI (AppliChem, Darmstadt, Germany). Cells were treated and 5.0 × 10^5^ harvested 6, 24, and 48 h after photodynamic treatment as described for the detection of lipid peroxidation. Cells were washed twice with PBS and fixed by ice-cold 70% (*v*/*v*) ethanol at 4 °C for 30 min. Fixed cells were centrifuged at 4.000 rpm at 4 °C for 10 min and resuspended in PBS, containing 25 µg/mL PI and 100 µg/mL RNase A (Carl Roth, Karlsruhe, Germany). After staining at room temperature for 30 min in the dark, cells were analyzed with a MACS Quant flow cytometer. For each sample, 10,000 events were counted and gated for the single cell population. The PI channel (λ_Ex/Em_ = 488/655–730 nm) was used for the detection of PI-positive cells. Data were analyzed with the MACS Quantify Software. After analysis, cells were assigned to either sub G_1_ (fragmented DNA, apoptotic), G_0_/G_1_, S, or G_2_/M phase.

### 4.7. Fluorescence Microscopy

Evaluation of Mitochondrial Membrane Potential (Δ*ψ*_M_)

Mitochondrial membrane potential (Δ*ψ*_m_) was detected with the BD MitoScreen JC-1 kit (BD Biosciences, San Diego, CA, USA) according to the kit instructions (modified for fluorescence microscopy). Briefly, living cells were washed with Assay Buffer within the wells of chamber slides 6 h after photodynamic treatment with the IC_90_ of mTHPC and stained with 2 µM JC-1 solution at 37 °C for 20 min. Afterwards, cells were washed twice with Assay Buffer and JC-1 monomers and aggregates were visualized using a Leica DMi8 fluorescence microscope (Leica, Munich, Germany) equipped with a 63× oil/1.4 NA objective. The disruption of active mitochondria is an early sign of apoptosis after oxidative stress induction [24,25]. The cationic JC-1 (5,5′,6,6′-tetrachloro-1,1′,3,3′-tetraethylbenzimidazolylcarbocyanine iodide) dye accumulates in mitochondria with normal or hyperpolarized Δ*ψ*_m_, leading to the formation of aggregates with a red fluorescence at 590 nm, whereas cytosolic JC-1 monomers display a green fluorescence at 527 nm. A decline in Δ*ψ*_m_ is thus indicated by a decrease in JC-1 aggregates, visualized by a decrease in red fluorescence [80]. Fluorescence images (1.392 × 1.040 px) of JC-1 monomers and aggregates were captured with the FITC filter cube (λ_Ex/Em_ = 460–500/512–542 nm) and the RHOD filter cube (λ_Ex/Em_ = 541–551/565–605 nm), respectively, and processed with the LAS X software (Leica, Munich, Germany). Solvent-treated and non-illuminated cells served as the negative control with active mitochondria. As a positive control, cells were treated with 50 µM carbonyl cyanide *m*-chloro phenyl hydrazone (CCCP; Alfa Aesar, Karlsruhe, Germany) at 37 °C for 5 min. CCCP is a well-known mitochondrial oxidative phosphorylation uncoupler [81].

### 4.8. Western Blotting

Cells were collected 6 (LC3-II) or 24 h (PARP, cas 3, LC3-II) after photodynamic treatment, washed twice with PBS and lysed with 100 µL ice-cold lysis buffer, containing 0.1 M NaCl, 0.1 M NaF, 0.2 mM Na_3_VO_4_ (AppliChem, Darmstadt, Germany), 5 mM EDTA, 1% (*v*/*v*) protease inhibitor cocktail (both Sigma-Aldrich, Munich, Germany) and 0.1% (*v*/*v*) Triton X-100 in 50 mM Tris buffer, pH 7.4. After ultrasonic treatment for 10 min, cells were centrifuged at 18.000 g at 4 °C for 10 min. For western blot analyses, equal amounts of total protein (10–30 µg) were heated to 95 °C for 5 min in Laemmli buffer [82], separated by SDS-PAGE and transferred to a polyvinylidene difluoride (PVDF) membrane (Bio-Rad, Karlsruhe, Germany). Membranes were blocked with 10% (w/v) non-fat milk in Tris buffer containing 0.05% (*v*/*v*) Tween-20 (Carl Roth, Karlsruhe, Germany) at room temperature for 1.5 h and afterwards incubated with the following primary rabbit antibodies at 4 °C overnight in Tris buffer supplemented with 0.05% (*v*/*v*) Tween-20 and 5% (w/v) BSA (Biomol, Hamburg, Germany); antibodies: PARP/cleaved PARP, (pro-)caspase 3, LC3-II, and GAPDH as loading control (all antibodies from Cell Signaling, Leiden, Netherlands). Afterwards, the membranes were incubated with goat anti-rabbit secondary IgG conjugated to horseradish peroxidase (Sigma-Aldrich, Munich, Germany) at room temperature for 4 h in the dark. Chemiluminescence was detected after incubation with enhanced chemiluminescence (ECL) substrate solution (Bio-Rad, Karlsruhe, Germany) for 2 min in the dark and visualized with an ECL imager (ChemoCam; Intas, Göttingen, Germany). Membranes were washed in between all steps with Tris buffer containing 20% (*v*/*v*) Tween-20 for three times. Semi-quantitative, densitometric western blot analysis has been done with LabImage 1D L340 software (Intas, Göttingen, Germany). In the case of LC3-II detection, an incubation with lysosomal protease inhibitors pepstatin A (100 µM; Sigma-Aldrich, Munich, Germany) and E-64d (10 µg/mL; Cayman Chemical, Ann Arbor, MI, USA) was carried out for 4 h before illuminating the cells. Furthermore, cells were incubated with phosphatidylinositol 3-kinase (PI3K) inhibitor wortmannin (2 µM; Sigma-Aldrich, Munich, Germany) for blockage of autophagy for 1 h before PDT. LC3-II levels were evaluated in the presence of inhibitors 24 h after illumination.

### 4.9. Statistical Analysis

Data were presented as means ± standard deviation (SD) of at least three independent experiments. Significant differences were detected by one-way or two-way ANOVA followed by Dunnett’s multiple comparisons test implemented by Prism 6 (GraphPad Software, La Jolla, CA, USA). A *p*-value < 0.05 was considered statistically significant.

## 5. Conclusions

In general, the overall phototoxic effects of mTHPC-PDT vary in dependency of concentration and time from cell line to cell line, suggesting that the cancer cells are not all dying by one defined mechanism, but rather succumb to an individual interplay of different cell death mechanisms. Triggering of oxidative stress by mTHPC-PDT was proven by a loss of mitochondrial membrane potential (Δ*ψ*_m_) and increased generation of ROS in all cell lines, although high light doses were required for enhanced ROS formation in some cases. However, lipid peroxidation (LPO) appeared to play only a minor role in most cell lines. Cellular death after high-dose mTHPC-PDT was mainly characterized by the induction of caspase-dependent apoptosis, which led to cleavage of PARP. The loss of Δ*ψ*_m_ confirmed the induction of apoptosis. Cell cycle analysis revealed that sooner or later DNA fragmentation occurred (increased sub G_1_ fraction), which was in some cell lines accompanied or preceded by an arrest in the G_2_/M cell cycle phase. At low-dose PDT, autophagy tends to have a pro-survival role, which counteracts apoptosis, while after high-dose PDT autophagy-associated apoptosis occurred. However, autophagy was just induced after either low- or high-dose PDT in most cell lines. Unregulated necrosis appeared to play only a minor role both after low- and high-dose PDT. Ideal dosage and incubation times in mTHPC-mediated PDT are rather individual for a specific tumor type, since the phototoxic effects vary widely in cells from different tissues. This study shows that general conclusions after PDT in vitro require testing on multiple cell lines to be reliable and that instead of applying one specific protocol for all cancer types, it would be advisable to individualize PS and light doses for the most effective outcome of a PDT treatment.

## Figures and Tables

**Table 1:** Established IC_50_ and IC_90_ values from MTT cell viability assay 24 h after incubation in the dark and post illumination with a light dose of 1.8 J/cm^2^, respectively.

	Dark					1.8 J/cm^2^				
	A-427	BHY	KYSE-70	RT-4	SISO	A-427	BHY	KYSE-70	RT-4	SISO
IC_50_ (µM)	0.6	1.0	1.0	1.8	1.0	0.02	0.06	0.06	0.1	0.06
IC_90_ (µM)	1.4	2.8	2.8	3.2	1.9	0.07	0.1	0.2	0.3	0.1

**Table 2:** Established % cytotoxicity as assessed by lactate dehydrogenase (LDH) release assay after treatment of cells with 0.1% (*v*/*v*, 6 h) and 0.01% (*v*/*v*, 24 h) Triton X-100 positive control after incubation in the dark and post illumination with a light dose of 1.8 J/cm^2^ (24 h) or 3.5 J/cm^2^ (6 h), respectively. Results as means ± SD from at least three independent experiments.

Triton X-100	LDH Release (% Cytotoxicity ± SD)									
	Dark					+Light				
	A-427	BHY	KYSE-70	RT-4	SISO	A-427	BHY	KYSE-70	RT-4	SISO
0.1%, 6 h	107.4	97.1	81.7	104.2	98.1	111.2	95.3	99.5	102.0	101.8
	±14.8	±3.8	±30.6	±10.2	±10.0	±23.4	±7.9	±18.7	±7.8	±10.8
0.01%, 24 h	59.7	57.9	66.5	57.7	70.3	45.2	51.1	63.2	47.5	75.3
	±13.3	±6.7	±12.7	±10.1	±14.9	±11.6	±2.8	±17.4	±5.4	±29.6

## Abbreviations

CCCP	Carbonyl Cyanide *m*-Chloro Phenyl Hydrazone
DCF	2′,7′-Dichlorofluorescein
DOXO	Doxorubicin
FBS	Fetal Bovine Serum
GP_X_	Glutathione Peroxidase
GSH	Glutathione
GSR	Glutathione-disulfide Reductase
H_2_DCF-DA	2′,7′-Dichlorodihydrofluorescein Diacetate
INT	2-(4-Iodophenyl)-3-(4-nitrophenyl)-5-phenyl-2*H*-tetrazolium Chloride
LDH	Lactate Dehydrogenase
LED	Light-emitting Diode
LPO	Lipid Peroxidation
mTHPC mTHPC-PDT	5,10,15,20-tetra(*m*-hydroxyphenyl)chlorin mTHPC induced Photodynamic Therapy
MTT	3-(4,5-Dimethyl-2-thiazolyl)-2,5-diphenyl-2*H*-tetrazolium Bromide
PARP	Poly(ADP-Ribose) Polymerase
PBS	Phosphate Buffered Saline
PDT	Photodynamic Therapy
PI	Propidium Iodide
PpIX	Protoporphyrin IX
PS	Photosensitizer
ROS	Reactive Oxygen Species
SC	Solvent-treated Control
*t*-BHP	*tert*-Butyl Hydroperoxide

## Appendix A

**Table A1:** Established % cellular viability as assessed by the MTT cell viability assay (with remaining cells from LDH release assay) after treatment of cells with 0.1% (*v*/*v*, 6 h) and 0.01% (*v*/*v*, 24 h) Triton X-100 positive control after incubation in the dark and post illumination with 1.8 J/cm^2^ (24 h) or 3.5 J/cm^2^ (6 h), respectively. Results as means ± SD from at least three independent experiments.

Triton X-100	% Cellular Viability ± SD									
	Dark					+Light				
	A-427	BHY	KYSE-70	RT-4	SISO	A-427	BHY	KYSE-70	RT-4	SISO
**0.1%, 6 h**	5.5	2.6	0	0.3	0.5	1.9	3.1	0.1	0.3	0.3
	±9.0	±3.9	+ 0.7	±0.3	±0.5	±2.9	±5.0	±0.6	±0.4	±0.2
**0.01%, 24 h**	1.7	2.6	5.8	0.2	3.2	3.0	2.5	5.1	0.3	2.4
	±2.7	±3.3	±5.2	±0.6	±2.4	±2.5	±3.3	±4.7	±0.4	±1.6

## References

[1] Lange C., Bednarski P.J. (2016). Photosensitizers for photodynamic therapy: Photochemistry in the service of oncology. Curr. Pharm. Des..

[2] Allison R.R., Sibata C.H. (2010). Oncologic photodynamic therapy photosensitizers: A clinical review. Photodiagn. Photodyn. Ther..

[3] Brown S.B., Brown E.A., Walker I. (2004). The present and future role of photodynamic therapy in cancer treatment. Lancet Oncol..

[4] Bonnett R., White R.D., Winfield U.J., Berenbaum M.C. (1989). Hydroporphyrins of the meso-tetra(hydroxyphenyl)porphyrin series as tumour photosensitizers. Biochem. J..

[5] De Visscher S.A., Dijkstra P.U., Tan I.B., Roodenburg J.L., Witjes M.J. (2013). mTHPC mediated photodynamic therapy (PDT) of squamous cell carcinoma in the head and neck: A systematic review. Oral Oncol..

[6] Senge O., Brandt J.C. (2011). Temoporfin (Foscan^®^, 5,10,15,20-tetra(m-hydroxyphenyl)chlorin)—A second-generation photosensitizer. Photochem. Photobiol..

[7] Marchal S., Dolivet G., Lassalle H.-P., Guillemin F., Bezdetnaya L. (2015). Targeted photodynamic therapy in head and neck squamous cell carcinoma: Heading into the future. Lasers Med. Sci..

[8] Hopkinson H.J., Vernon D.I., Brown S.B. (1999). Identification and partial characterization of an unusual distribution of the photosensitizer meta-tetrahydroxyphenyl chlorin (temoporfin) in human plasma. Photochem. Photobiol..

[9] Civantos F.J., Karakullukcu B., Biel M., Silver C.E., Rinaldo A., Saba N.F., Ferlito A. (2018). A review of photodynamic therapy for neoplasms of the head and neck. Adv. Ther..

[10] Nelke K.H., Pawlak W., Leszczyszyn J., Gerber H. (2014). Photodynamic therapy in head and neck cancer. Postepy Hig. Med. Dosw..

[11] Reuther T., Kubler A.C., Zillmann U., Flechtenmacher C., Sinn H. (2001). Comparison of the in vivo efficiency of Photofrin II-, mTHPC-, mTHPC-PEG- and mTHPCnPEG-mediated PDT in a human xenografted head and neck carcinoma. Lasers Surg. Med..

[12] Meier D., Botter S.M., Campanile C., Robl B., Gräfe S., Pellegrini G., Fuchs B. (2017). Foscan and foslip based photodynamic therapy in osteosarcoma in vitro and in intratibial mouse models. Int. J. Cancer.

[13] Gheewala T., Skwor T., Munirathinam G. (2017). Photosensitizers in prostate cancer therapy. Oncotarget.

[14] Wachowska M., Osiak A., Muchowicz A., Gabrysiak M., Domagala A., Kilarski W.W., Golab J. (2016). Investigation of cell death mechanisms in human lymphatic endothelial cells undergoing photodynamic therapy. Photodiagn. Photodyn. Ther..

[15] François A., Marchal S., Guillemin F., Bezdetnaya L. (2011). mTHPC-based photodynamic therapy induction of autophagy and apoptosis in cultured cells in relation to mitochondria and endoplasmic reticulum stress. Int. J. Oncol..

[16] Marchal S., François A., Dumas D., Guillemin F., Bezdetnaya L. (2007). Relationship between subcellular localisation of Foscan and caspase activation in photosensitised MCF-7 cells. Br. J. Cancer.

[17] Kiesslich T., Berlanda J., Plaetzer K., Krammer B., Berr F. (2007). Comparative characterization of the efficiency and cellular pharmacokinetics of Foscan- and Foslip-based photodynamic treatment in human biliary tract cancer cell lines. Photochem. Photobiol. Sci..

[18] Chan F.K., Moriwaki K., De Rosa M.J. (2013). Detection of necrosis by release of lactate dehydrogenase activity. Methods Mol. Biol..

[19] Kessel D., Pandey R.K., Kessel D., Dougherty T.J. (2016). PDT: Death and survival pathways. Handbook of Photodynamic Therapy: Updates on Recent Applications of Porphyrin-Based Compounds.

[20] Halliwell B., Whiteman M. (2004). Measuring reactive species and oxidative damage in vivo and in cell culture: How should you do it and what do the results mean?. Br. J. Pharmacol..

[21] LeBel C.P., Ischiropoulos H., Bondy S.C. (1992). Evaluation of the probe 2’,7’-dichlorofluorescin as an indicator of reactive oxygen species formation and oxidative stress. Chem. Res. Toxicol..

[22] Raudsepp P., Brüggemann D.A., Andersen M.L. (2014). Detection of radicals in single droplets of oil-in-water emulsions with the lipophilic fluorescent probe BODIPY665/676 and confocal laser scanning microscopy. Free Radic. Biol. Med..

[23] Drummen G.P.C., van Liebergen L.C.M., op den Kamp J.A.F., Post J.A. (2002). C11-BODIPY581/591, an oxidation-sensitive fluorescent lipid peroxidation probe: (micro)spectroscopic characterization and validation of methodology. Free Radic. Biol. Med..

[24] Richter C., Wyss P., Tadir Y., Tromberg B.J., Haller U. (2000). Mitochondria as targets for the induction of apoptosis in photodynamic therapy. Photomedicine in Gynecology and Reproduction.

[25] Satoh T., Enokido Y., Aoshima H., Uchiyama Y., Hatanaka H. (1997). Changes in mitochondrial membrane potential during oxidative stress-induced apoptosis in PC12 cells. J. Neurosci. Res..

[26] Castano A.P., Demidova T.N., Hamblin M.R. (2005). Mechanisms in photodynamic therapy: Part two—Cellular signaling, cell metabolism and modes of cell death. Photodiagn. Photodyn. Ther..

[27] Oleinick N.L., Morris R.L., Belichenko I. (2002). The role of apoptosis in response to photodynamic therapy: What, where, why, and how. Photochem. Photobiol..

[28] Yu S.-W., Andrabi S.A., Wang H., Kim N.S., Poirier G.G., Dawson T.M., Dawson V.L. (2006). Apoptosis-inducing factor mediates poly(ADP-ribose) (PAR) polymer-induced cell death. Proc. Natl. Acad. Sci. USA.

[29] Chaitanya G.V., Steven A.J., Babu P.P. (2010). PARP-1 cleavage fragments: Signatures of cell-death proteases in neurodegeneration. Cell Commun. Signal..

[30] Nagata S., Nagase H., Kawane K., Mukae N., Fukuyama H. (2003). Degradation of chromosomal DNA during apoptosis. Cell Death Differ..

[31] Klionsky D.J., Abdelmohsen K., Abe A., Abedin M.J., Abeliovich H., Arozena A.A., Zughaier S.M. (2016). Guidelines for the use and interpretation of assays for monitoring autophagy (3rd edition). Autophagy.

[32] Mizushima N., Yoshimori T. (2007). How to interpret LC3 immunoblotting. Autophagy.

[33] Blommaart E.F., Krause U., Schellens J.P., Vreeling-Sindelarova H., Meijer A.J. (1997). The phosphatidylinositol 3-kinase inhibitors wortmannin and LY294002 inhibit autophagy in isolated rat hepatocytes. Eur. J. Biochem..

[34] Berlanda J., Kiesslich T., Engelhardt V., Krammer B., Plaetzer K. (2010). Comparative in vitro study on the characteristics of different photosensitizers employed in PDT. J. Photochem. Photobiol. B.

[35] Abdulrehman G., Xv K., Li Y., Kang L. (2018). Effects of meta-tetrahydroxyphenylchlorin photodynamic therapy on isogenic colorectal cancer SW480 and SW620 cells with different metastatic potentials. Lasers Med. Sci..

[36] Löw K., Knobloch T., Wagner S.J., Wiehe A., Engel A., Langer K., von Briesen H. (2011). Comparison of intracellular accumulation and cytotoxicity of freemTHPC andmTHPC-loaded PLGA nanoparticles in human colon carcinoma cells. Nanotechnology.

[37] Agostinis P., Berg K., Cengel K.A., Foster T.H., Girotti A.W., Gollnick S.O., Golab J. (2011). Photodynamic therapy of cancer: An update. CA Cancer J. Clin..

[38] Girotti A.W. (2001). Photosensitized oxidation of membrane lipids: Reaction pathways, cytotoxic effects, and cytoprotective mechanisms. J. Photochem. Photobiol. B.

[39] Bilski P., Belanger A.G., Chignell C.F. (2002). Photosensitized oxidation of 2’,7’-dichlorofluorescin: Singlet oxygen does not contribute to the formation of fluorescent oxidation product 2’,7’-dichlorofluorescein. Free Radic. Biol. Med..

[40] Redmond R.W., Gamlin J.N. (1999). A compilation of singlet oxygen yields from biologically relevant molecules. Photochem. Photobiol..

[41] Bonnett R., Charlesworth P., Djelal B.D., Foley S., McGarvey D.J., Truscott T.G. (1999). Photophysical properties of 5,10,15,20-tetrakis(m-hydroxyphenyl)porphyrin (m-THPP), 5,10,15,20-tetrakis(m-hydroxyphenyl)chlorin (m-THPC) and 5,10,15,20-tetrakis(m-hydroxyphenyl)bacteriochlorin (m-THPBC): A comparative study. J. Chem. Soc. Perkin Trans. 2.

[42] Bracht K., Boubakari, Grünert R., Bednarski P.J. (2006). Correlations between the activities of 19 anti-tumor agents and the intracellular glutathione concentrations in a panel of 14 human cancer cell lines: Comparisons with the National Cancer Institute data. Anticancer Drugs.

[43] Ehrenberg B., Gross E., Nitzan Y., Malik Z. (1993). Electric depolarization of photosensitized cells: Lipid vs. protein alterations. Biochim. Biophys. Acta.

[44] Gaullier J.M., Valla A., Bazin M., Giraud M., Dubertret L., Santus R. (1997). N-conjugates of 2,5-disubstituted pyrrole and glutathione. Evaluation of their potency as antioxidants against photosensitization of NCTC 2544 keratinocytes by excess endogenous protoporphyrin IX. J. Photochem. Photobiol. B.

[45] Klein S.D., Walt H., Richter C. (1997). Photosensitization of isolated rat liver mitochondria by tetra(m-hydroxyphenyl)chlorin. Arch. Biochem. Biophys..

[46] Melnikova V.O., Bezdetnaya L.N., Potapenko A.Y., Guillemin F. (1999). Photodynamic properties of meta-tetra(hydroxyphenyl)chlorin in human tumor cells. Radiat. Res..

[47] Kirveliene V., Prasmickaite L., Kadziauskas J., Bonnett R., Djelal B.D., Juodka B. (1997). Post-exposure processes in Temoporfin-photosensitized cells in vitro: Reliance on energy metabolism. J. Photochem. Photobiol. B.

[48] Marchal S., Fadloun A., Maugain E., D’Hallewin M.A., Guillemin F., Bezdetnaya L. (2005). Necrotic and apoptotic features of cell death in response to Foscan photosensitization of HT29 monolayer and multicell spheroids. Biochem. Pharmacol..

[49] Marchal S., Bezdetnaya L., Guillemin F. (2004). Modality of cell death induced by Foscan-based photodynamic treatment in human colon adenocarcinoma cell line HT29. Biochemistry (Mosc.).

[50] Yow C.M.N., Mak N.K., Leung A.W.N., Huang Z. (2009). Induction of early apoptosis in human nasopharyngeal carcinoma cells by mTHPC-mediated photocytotoxicity. Photodiagn. Photodyn. Ther..

[51] Segawa K., Nagata S. (2015). An apoptotic ‘me’ signal: Phosphatidylserine exposure. Trends Cell Biol..

[52] Segawa K., Kurata S., Yanagihashi Y., Brummelkamp T.R., Matsuda F., Nagata S. (2014). Caspase-mediated cleavage of phospholipid flippase for apoptotic phosphatidylserine exposure. Science.

[53] Mandal D., Moitra P.K., Saha S., Basu J. (2002). Caspase 3 regulates phosphatidylserine externalization and phagocytosis of oxidatively stressed erythrocytes. FEBS Lett..

[54] Boucher D., Blais V., Denault J.-B. (2012). Caspase-7 uses an exosite to promote poly(ADP ribose) polymerase 1 proteolysis. Proc. Natl. Acad. Sci. USA.

[55] D’Amours D., Sallmann F.R., Dixit V.M., Poirier G.G. (2001). Gain-of-function of poly(ADP-ribose) polymerase-1 upon cleavage by apoptotic proteases: Implications for apoptosis. J. Cell Sci..

[56] Puig B., Tortosa A., Ferrer I. (2001). Cleaved caspase-3, caspase-7 and poly (ADP-ribose) polymerase are complementarily but differentially expressed in human medulloblastomas. Neurosci. Lett..

[57] Wyrsch P., Blenn C., Bader J., Althaus F.R. (2012). Cell death and autophagy under oxidative stress: Roles of poly(ADP-ribose) polymerases and Ca^2+^. Mol. Cell. Biol..

[58] Zhang N., Chen Y., Jiang R., Li E., Chen X., Xi Z., Jiang X. (2011). PARP and RIP 1 are required for autophagy induced by 11’-deoxyverticillin A, which precedes caspase-dependent apoptosis. Autophagy.

[59] Luo X., Kraus W.L. (2012). On PAR with PARP: Cellular stress signaling through poly(ADP-ribose) and PARP-1. Genes Dev..

[60] Herceg Z., Wang Z.-Q. (1999). Failure of poly(ADP-ribose) polymerase cleavage by caspases leads to induction of necrosis and enhanced apoptosis. Mol. Cell. Biol..

[61] Bacellar I.O., Tsubone T.M., Pavani C., Baptista M.S. (2015). Photodynamic efficiency: From molecular photochemistry to cell death. Int. J. Mol. Sci..

[62] Mroz P., Yaroslavsky A., Kharkwal G.B., Hamblin M.R. (2011). Cell death pathways in photodynamic therapy of cancer. Cancers.

[63] Gharehbaghi K., Kubin A., Grusch M., Gharehbaghi-Schnell E., Wierrani F., Jayaram H.N., Szekeres T. (2000). Photodynamic action of meta-tetrahydroxyphenylchlorin (mTHPC) on an ovarian cancer cell line. Anticancer Res..

[64] Wlodkowic D., Telford W., Skommer J., Darzynkiewicz Z., Darzynkiewicz Z., Holden E., Orfao A., Telford W., Wlodkowic D. (2011). Apoptosis and beyond: Cytometry in studies of programmed cell death. Methods in Cell Biology: Recent Advances in Cytometry, Part B.

[65] Kitazumi I., Tsukahara M. (2011). Regulation of DNA fragmentation: The role of caspases and phosphorylation. FEBS J..

[66] Chaudhuri A.R., Nussenzweig A. (2017). The multifaceted roles of PARP1 in DNA repair and chromatin remodelling. Nat. Rev. Mol. Cell Biol..

[67] Claybon A., Karia B., Bruce C., Bishop A.J.R. (2010). PARP1 suppresses homologous recombination events in mice in vivo. Nucleic Acids Res..

[68] Kukcinaviciute E., Sasnauskiene A., Dabkeviciene D., Kirveliene V., Jonusiene V. (2017). Effect of mTHPC-mediated photodynamic therapy on 5-fluorouracil resistant human colorectal cancer cells. Photochem. Photobiol. Sci..

[69] Teiten M.H., Bezdetnaya L., Morliere P., Santus R., Guillemin F. (2003). Endoplasmic reticulum and Golgi apparatus are the preferential sites of Foscan^®^ localisation in cultured tumour cells. Br. J. Cancer.

[70] Chen J.Y., Mak N.K., Yow C.M., Fung M.C., Chiu L.C., Leung W.N., Cheung N.H. (2000). The binding characteristics and intracellular localization of temoporfin (mTHPC) in myeloid leukemia cells: Phototoxicity and mitochondrial damage. Photochem. Photobiol..

[71] Inguscio V., Panzarini E., Dini L. (2012). Autophagy contributes to the death/survival balance in cancer photodynamic therapy. Cells.

[72] Andrzejak M., Price M., Kessel D.H. (2011). Apoptotic and autophagic responses to photodynamic therapy in 1c1c7 murine hepatoma cells. Autophagy.

[73] Reiners J.J., Agostinis P., Berg K., Oleinick N.L., Kessel D. (2010). Assessing autophagy in the context of photodynamic therapy. Autophagy.

[74] Sasnauskiene A., Kadziauskas J., Vezelyte N., Jonusiene V., Kirveliene V. (2009). Apoptosis, autophagy and cell cycle arrest following photodamage to mitochondrial interior. Apoptosis.

[75] Pieslinger A., Plaetzer K., Oberdanner C.B., Berlanda J., Mair H., Krammer B., Kiesslich T. (2006). Characterization of a simple and homogeneous irradiation device based on light-emitting diodes: A possible low-cost supplement to conventional light sources for photodynamic treatment. Med. Laser Appl..

[76] Lange C., Bednarski P.J. (2018). Evaluation for synergistic effects by combinations of photodynamic therapy (PDT) with temoporfin (mTHPC) and Pt(II) complexes carboplatin, cisplatin or oxaliplatin in a set of five human cancer cell lines. Int. J. Mol. Sci..

[77] Haidara K., Morel I., Abalea V., Gascon Barre M., Denizeau F. (2002). Mechanism of tert-butylhydroperoxide induced apoptosis in rat hepatocytes: Involvement of mitochondria and endoplasmic reticulum. Biochim. Biophys. Acta.

[78] Rebbaa A., Zheng X., Chou P.M., Mirkin B.L. (2003). Caspase inhibition switches doxorubicin-induced apoptosis to senescence. Oncogene.

[79] Skladanowski A., Konopa J. (1993). Adriamycin and daunomycin induce programmed cell death (apoptosis) in tumour cells. Biochem. Pharmacol..

[80] Perelman A., Wachtel C., Cohen M., Haupt S., Shapiro H., Tzur A. (2012). JC-1: Alternative excitation wavelengths facilitate mitochondrial membrane potential cytometry. Cell Death Dis..

[81] Zhang Y.Q., Shen X., Xiao X.L., Liu M.Y., Li S.L., Yan J., Dong D.L. (2016). Mitochondrial uncoupler carbonyl cyanide m-chlorophenylhydrazone induces vasorelaxation without involving KATP channel activation in smooth muscle cells of arteries. Br. J. Pharmacol..

[82] Laemmli U.K. (1970). Cleavage of structural proteins during the assembly of the head of bacteriophage T4. Nature.

